# Phosphatase inhibitor PPP1R11 modulates resistance of human T cells toward Treg‐mediated suppression of cytokine expression

**DOI:** 10.1002/JLB.2A0618-228R

**Published:** 2019-03-18

**Authors:** Rubin N. Joshi, Sunjay Jude Fernandes, Ming‐Mei Shang, Narsis A. Kiani, David Gomez‐Cabrero, Jesper Tegnér, Angelika Schmidt

**Affiliations:** ^1^ Unit of Computational Medicine Center for Molecular Medicine Department of Medicine Solna Karolinska University Hospital and Science for Life Laboratory Karolinska Institutet Stockholm Sweden; ^2^ Biological and Environmental Sciences and Engineering Division Computer Electrical and Mathematical Sciences and Engineering Division King Abdullah University of Science and Technology (KAUST) Thuwal Saudi Arabia; ^3^ Division of Rheumatology Department of Medicine Solna Karolinska University Hospital Karolinska Institutet Stockholm Sweden; ^4^ Mucosal and Salivary Biology Division King's College London Dental Institute London United Kingdom; ^5^ Translational Bioinformatics Unit NavarraBiomed Departamento de Salud‐Universidad Pública de Navarra Pamplona Navarra Spain

**Keywords:** regulatory T cell, CD4 T cell, immune suppression, T cell resistance, phosphatase, immunotherapy

## Abstract

Regulatory T cells (Tregs) act as indispensable unit for maintaining peripheral immune tolerance mainly by regulating effector T cells. T cells resistant to suppression by Tregs pose therapeutic challenges in the treatment of autoimmune diseases, while augmenting susceptibility to suppression may be desirable for cancer therapy. To understand the cell intrinsic signals in T cells during suppression by Tregs, we have previously performed a global phosphoproteomic characterization. We revealed altered phosphorylation of protein phosphatase 1 regulatory subunit 11 (PPP1R11; Inhibitor‐3) in conventional T cells upon suppression by Tregs. Here, we show that silencing of PPP1R11 renders T cells resistant toward Treg‐mediated suppression of TCR‐induced cytokine expression. Furthermore, whole‐transcriptome sequencing revealed that PPP1R11 differentially regulates not only the expression of specific T cell stimulation‐induced cytokines but also other molecules and pathways in T cells. We further confirmed the target of PPP1R11, PP1, to augment TCR‐induced cytokine expression. In conclusion, we present PPP1R11 as a novel negative regulator of T cell activation‐induced cytokine expression. Targeting PPP1R11 may have therapeutic potential to regulate the T cell activation status including modulating the susceptibility of T cells toward Treg‐mediated suppression, specifically altering the stimulation‐induced T cell cytokine milieu.

## Graphical abstract

Insights into a novel role of phosphatase inhibitor PPP1R11 in inducing resistance toward Treg‐mediated suppression of T cell activation‐induced cytokines in conventional T cells.

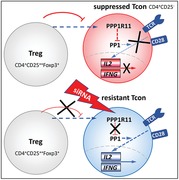

**Abbreviations**
AKT: AKT serine/threonine kinase 1 (protein kinase B)
BRAF: murine sarcoma viral oncogene homolog B
CD: cluster of differentiation
CFSE: carboxyfluorescein succinimidyl ester
DEG: differentially expressed gene
HEG: highly expressed gene
GO: gene ontology
Iono: ionomycin
LCK: leukocyte specific protein kinase
LEG: lowly expressed gene
LEPR: leptin receptor
MAP3K7CL: MAP3K7 C‐terminal like
MYB: V‐myb avian myeloblastosis viral oncogene homolog
PDPK1: phosphoinositide dependent protein kinase 1
PI4K2B: phosphatidylinositol 4‐kinase type 2 beta
PIP: PP1 interacting protein
PLA2G6: phospholipase A2, group VI
PLAU: plasminogen activator, urokinase
PP1: protein phosphatase 1
PPP1R11: protein phosphatase 1 regulatory subunit 11
PTPN22: protein Tyrosine Phosphatase, Non‐Receptor Type 22
RRAS2: related RAS viral (r‐ras) oncogene homolog 2
SLAT: SWAP‐70‐like adaptor protein of T Cells
Tcon: conventional T cell (CD4^+^, CD25^−^)
Treg: regulatory T cell

## 1 INTRODUCTION

CD4‐expressing T cells (CD4 T cells) are crucial in shaping the course of immune responses. Regulatory T cells (Tregs), a subtype of CD4 T cells, control the activation status of many different immune cells including other (conventional) CD4 T cells to enforce peripheral tolerance.^1^ Breach of this check and balance machinery resulting from a deficit in Treg number or functionality has been clearly shown to contribute to the pathogenesis of autoimmune diseases, allergies, and chronic inflammatory diseases.^2^, ^3^ On the opposite end of the spectrum, unwarranted immune suppression by Tregs is among the causes of immune evasion in cancer^4^ and pathogenic diseases. Several therapeutic approaches to target Tregs in autoimmune diseases^5^ and cancers^4^, ^6^, ^7^ have shown remarkable progress in recent years. However, it is alarming to realize that in several autoimmune diseases, there have been multiple reported cases of resistance of T cell toward Treg‐mediated suppression.^3^, ^8^, ^9^ We have recently performed the first global analysis to systematically study signaling molecules affected by Treg‐mediated suppression in conventional T cells (Tcons) by phosphoproteomics.^10^ These identified phosphoproteins bridge knowledge gaps existing regarding signaling cascades of suppressed T cells. These results can be helpful to understand T cell responses toward suppression by Tregs and hence the cell‐intrinsic causes of resistance toward Treg‐mediated suppression.

T cell receptor (TCR) stimulation in presence of co‐stimulation via CD28 signaling leads to activation of Src family kinases such as leukocyte specific protein kinase (LCK) and FYN Proto‐Oncogene (FYN), which phosphorylate intracellular CD3 residues, ZAP‐70, and other substrates. This initiates signaling events leading to activation of transcription factors such as AP‐1, NF‐κB, and NFAT. These transcription factors are crucial for expression of genes required for T cell function and survival such as activatory cytokines and other immunostimulatory molecules.^11^, ^12^ Aberration in the molecules involved in this signaling cascade of T cells may induce resistance toward suppression by Tregs. We and others have shown that Treg‐mediated rapid suppression of T cells is mediated via modulation of NFAT, AP‐1, and/or NF‐κB pathways with differences depending on the particular cell types, stimulation, and readouts used.^13^, ^14^, ^15^ Furthermore, clinical and experimental studies have reported the involvement of PI3K and protein kinase B (AKT; that are downstream of CD28 signaling) to be involved in inducing resistance in T cells.^8^, ^16^, ^17^, ^18^, ^19^, ^20^ Given these studies, Tregs seem to affect multiple signaling pathways in target T cells in a context‐dependent manner. The mechanisms by which Tregs affect these pathways have been understood only partly. We demonstrated that inhibition of calcium signaling with involvement of the phosphoprotein DEF6 (SLAT) was causative for NFAT inhibition.^10^, ^13^ Although artificial raise of calcium concentrations could also abrogate NF‐κB suppression due to crosstalk of these signaling pathways when strong calcium signaling was introduced,^13^ it is plausible that Tregs also directly affect unknown signaling molecules within the classical NF‐κB pathway. Furthermore, under which conditions and through which mechanisms Tregs inhibit AP‐1 activation remains unknown. Thus, to further elucidate these mechanistic aspects, we here specifically inspected our previously generated phosphoproteomics data^10^ for potential signaling modulators that control phosphorylation, such as kinases or phosphatases and their regulators.

Interestingly, we observed the phosphatase regulator, PPP1R11 (also called Inhibitor‐3), to be phosphorylated upon T cell activation and conversely dephosphorylated upon Treg‐mediated suppression of T cells.^10^ Due to extensive involvement of kinases and phosphatases in T cell signaling, PPP1R11 is likely to be a novel mediator of T cell signaling and resistance toward Treg‐mediated suppression.

PPP1R11 is a regulatory subunit of the protein phosphatase 1 (PP1) holoenzyme and functions as a potent inhibitor of PP1.^21^ PP1 is the most common of the eukaryotic phosphatase with a unique property of existing as more than 650 complexes arising from combinations of other subunits and PP1‐interacting proteins (PIP).^22^, ^23^ Substrate specificity of PP1 catalytic subunits, which themselves are relatively unspecific as compared to those of kinases, is modulated by noncatalytic subunits. Hence, therapeutic targeting of these noncatalytic subunits may offer a unique opportunity for context‐specific modulation of PP1. Importantly, PP1A has been recently shown to be involved in T cell activation and cytokine expression via augmenting TCR‐induced NF‐κB activation without affecting classical upstream NF‐κB signaling molecules.^24^, ^25^ PPP1R11 itself has been shown to be involved in apoptosis and cell cycle regulation chiefly by regulating the conformation of PP1 and affecting its interaction with PIPs rather than regulating gene expression of PP1.^26^, ^27^, ^28^

The effect of PPP1R11 on immune cells has not been widely studied. To understand the role of PPP1R11 in T cell biology, we here performed siRNA‐mediated silencing of PPP1R11 in primary human T cells, thereby removing the inhibitor of PP1 and unleashing PP1 activity. We observed that loss of PPP1R11 induced resistance toward Treg‐mediated suppression in T cells as measured by gene and protein expression of T cell stimulation‐induced cytokines IL‐2 and IFN‐γ. Loss of PPP1R11 did not cause any significant difference in the proliferation pattern of T cells while PPP1R11 silencing induced significant upregulation of *IL2, IFNG*, and other TCR‐stimulation‐induced cytokines. We provide insights on the global effect of PPP1R11 on shaping T cell signaling by performing RNAseq analysis on PPP1R11‐silenced and control T cells. RNAseq analyses indicate that PPP1R11 may affect pathways involving phosphatidylinositol signaling, possibly MAPK‐AKT, and NF‐κB pathways. Yet targeted analyses of major known signaling molecules involved in TCR‐induced cytokine expression did not unveil the mechanistic TCR signaling target of PPP1R11. Instead, inhibiting the major phosphatase target of PPP1R11, PP1, affected expression of the same cytokines. Taken together, we propose a novel function of PPP1R11 as a negative regulator of T cell activation‐induced cytokine expression and modulator of susceptibility of T cells toward Treg‐mediated suppression.

## 2 MATERIALS AND METHODS

### 2.1 Ethics statement

Buffy coats from anonymized healthy human donors purchased from the Karolinska University Hospital (Karolinska Universitetssjukhuset, Huddinge, Sweden) were used freshly for isolation of PBMCs. Research was performed according to the national Swedish ethical regulations (ethical review act, SFS number 2003:460). Ethical permit for the experiments was obtained from the Regional Ethical Review Board in Stockholm (Regionala etikprövningsnämnden i Stockholm), Sweden (approval number: 2013/1458‐31/1).

### 2.2 Preparation of human Tcons and Tregs

PBMCs were purified from fresh buffy coats by gradient centrifugation using Ficoll‐Paque Plus (GE Healthcare). Monocyte depletion was then performed by plastic adherence in RPMI 1640 medium containing 10% FCS (Invitrogen). Blood from HLA‐A2+ donors was used to isolate Tregs and Tcons, and blood from HLA‐A2− donors was used to isolate responder Tcons whenever Tcon:Treg coculture and subsequent coculture separation was involved. Before Treg isolation, PBMCs were rested overnight at 4°C, or directly used for magnetic‐activated cell sorting (MACS) isolation. From HLA‐A2+ donors, we first isolated CD25^high^ cells with CD25‐specific MACS beads (2 µl per 10^7^ cells, Miltenyi Biotec; cat. no. 130‐092‐983) as described previously.^13^ These Tregs were pre‐activated overnight with covalently plate‐bound Ab against CD3 as described.^10^ “Untouched” CD4^+^CD25^−^ control Tcons were isolated from the Treg‐depleted fraction using the CD4^+^ T cell Isolation Kit II, human (Miltenyi Biotec), and were additionally depleted from CD25^+^ cells with CD25‐specific MACS beads (6 µl per 10^7^ cells). Responder Tcons from HLA‐A2^−^ donors, or Tcons without HLA‐A2 determination for experiments, which do not involve Tregs, were isolated in a similar way, using 8 µl CD25 beads per 10^7^ cells for CD25 depletion instead in a single step.

Cell purity of all MACS‐isolated cells was assessed by flow cytometry. Cells were counted in trypan blue solution using a Countess Automated Cell Counter (Life Technologies) and viability was determined by trypan blue stain. Tcons and Tregs were cultured at 5% CO_2_/37°C in serum‐free X‐VIVO15 medium (Lonza) containing 1% GlutaMAX (Invitrogen).

### 2.3 Nucleofection

For experiments involving use of siRNA, 5–12 × 10^6^ CD25^−^CD4^+^ T cells from individual donors were resuspended in 100 µl of Nucleofection buffer solution for human primary T cells (Nucleofector Kits for Human T Cells, Lonza) containing 2 µM of ON‐TARGETplus PPP1R11 siRNA pools or ON‐TARGETplus nontargeting control pool (both Dharmacon, GE Healthcare). To deconvolute the effect of the siRNA pool, confirmation experiments were also performed with 2 individual PPP1R11 siRNAs (contained in the pool of 4 siRNAs above), which are denoted as PPP1R11‐07 and PPP1R11‐08. The cells were transfected using program U‐014 of the Amaxa Nucleofector™ 2b device using the manufacturer's recommendations. Following nucleofection, the cells were transferred to prewarmed X‐VIVO 15 medium and incubated for 4.5 days at 5% CO_2_/37°C unless otherwise mentioned. The medium was changed once following 5 h of incubation. The cells were either directly frozen for studying the unstimulated state or stimulated with particular stimulations for specific time periods depending on the nature of further analyses.

### 2.4 Coculture setup and T cell stimulation

For short term mRNA studies, HLA‐A2+ Tregs (pre‐activated and subsequently pooled from 1–3 donors to obtain sufficient cell numbers wherever necessary) and HLA‐A2+ Tcons (control) were labeled with FITC‐conjugated Ab against HLA‐A2 and FITC‐specific microbeads (Miltenyi Biotec), whereas HLA‐A2‐ Tcons (responder Tcons) were left untreated. Before setup of cocultures and stimulation, all cells were thoroughly washed in X‐VIVO 15 medium and resuspended in the same medium. Cocultures of HLA‐A2‐ responder Tcons (single donors) with either HLA‐A2^+^ Tregs or control HLA‐A2^+^ Tcons were set up in a 1:1 ratio. Cells were pre‐cocultured for 85 min and then stimulated. T cells were stimulated with soluble Ab against CD3 (0.2 µg/ml, clone OKT3, BioLegend, LEAF grade, cat. no. 317315), Ab against CD28 (2 µg/ml, clone 15E8, Miltenyi Biotec, functional grade, cat. no. 130‐093‐375), and goat anti‐mouse Ig Ab as a cross‐linker (2 µg/ml, Southern Biotech, cat. no. 1010‐01) mimicking TCR and co‐stimulation. As control for all allogenic cocultures, HLA‐A2‐Tcons were left unstimulated, or stimulated alone (without allogeneic cell coculture, at the same final cell density and number). For experiments without Tregs, T cells from single donors were stimulated in the same way unless specifically stated. Cells were stimulated for 5 min for protein studies and for 3 h for RNA studies respectively at 37°C and 5% CO_2_. Alternatively, T cells were stimulated with PMA (10 ng/ml; Sigma–Aldrich) and ionomycin (Iono; 375 ng/ml; Sigma–Aldrich) for 5 min to 3 h. Stimulation was then stopped with ice‐cold MACS buffer (0.5% (w/v) HSA, 2 mM EDTA, in PBS), and wherever applicable, the different cell populations were separated on the basis of HLA‐A2 expression by passing the cells over an LS column (Miltenyi Biotec) on ice; control cells including unstimulated cells were treated and passed over LS columns in the same way. HLA‐A2‐ Tcons (flow through from the columns; >96% pure) were used for subsequent mRNA and protein analyses. After stimulation (and coculture separation where applicable), cells were centrifuged (450 × *g*, 8 min, 4°C) and supernatant was removed. For protein studies, cells were further washed twice with 1 ml ice‐cold PBS each (1000 × *g*, 5 min, 4°C) and the supernatant was removed completely before use of cell pellets (see below). For RNA studies, cell pellets were stored at –20°C before analysis (see below).

For long‐term stimulations, cells were stimulated for 4 to 6 days with plate‐bound anti‐CD3 Ab (same manufacturer as earlier mentioned) and soluble anti‐CD28 Ab (1 µg/ml, BioLegend, LEAF grade, cat. no. 302923) as follows. U well plates were coated with 65 µl of 5 µg/ml anti‐CD3 Ab in PBS and incubated overnight at 4˚C followed by 2 washes with PBS prior to addition of culture medium including anti‐CD28 Ab. For long‐term stimulations including Tcon:Treg cocultures involving measurement of cytokine concentrations in the supernatant, similar setup as short‐term coculture was used except coculture separation and hence HLA‐A2 determination was not performed, given that Tregs are anergic in vitro in terms of IL‐2 and IFN‐γ secretion. Further the cocultures were immediately activated without the need of pre‐coculture, since Tregs will be activated long enough within the long‐term assay. Cultures of Tcons without Tregs were taken as positive control and cultures of Tregs alone were taken as negative controls. The negative control was used to ensure that the cytokine reading was not affected by cytokines secreted from Tregs.

### 2.5 Phosphoproteomics

T cell samples were chemically labeled using stable isotope dimethyl labeling prior to phosphopeptide enrichment with Ti^4+^ IMAC and analyzed with LC–MS. Detailed procedure for LC–MS along with data analysis process have been described earlier.^10^, ^29^, ^30^

### 2.6 RNA preparation and quantitative RT‐PCR

Total RNA for quantitative RT‐PCR (qRT‐PCR) experiments was isolated from frozen cell pellets using the RNAqueous Micro Kit (Ambion), quantified with a Nanodrop 2000 (Thermo Scientific), and cDNA was prepared using the SuperScript VILO cDNA Synthesis Kit (Invitrogen) according to the manufacturer's instructions. Relative mRNA levels of *GAPDH*, *RPL13A*, *IFNG*, *IL2, PPP1R11, PP1*, *IL2RA*, *CD69*, or *CTLA4* were quantified using Taqman probes (Applied Biosystems best coverage probes, all with FAM reporter) with the Taqman gene expression master mix (Applied Biosystems) or with SYBR Green primers (Sigma–Aldrich; primer sequences as described before^10^ or as follows: *CTLA4*: Frw: TCC TGT TTT TTC TTC TCT TCA TCC C, Rev: CCA CGT GCA TTG CTT TGC) with the Power SYBR Green PCR Master Mix (Applied Biosystems). Samples were measured on a StepOne plus detector system (Applied Biosystems). The relative mRNA expression was determined by normalization to *RPL13A* and/or *GAPDH*. Results are presented as fold induction compared to mRNA amounts of control samples (unstimulated or control siRNA‐treated samples from the same donor as indicated in individual figure legends), which were set to 1. Fold expression was calculated using the ΔΔCt method according to the following formula (Ct is the threshold cycle value):

$$\text{Relative}\;\text{mRNA}\;\text{expression}=2^{-(\text{Ct}\;\text{of}\;\text{gene}\;\text{of}\;\text{interest}-\text{Ct}\;\text{of}\;\text{RPL13A}\;\text{or}\;\text{GAPDH})}$$

### 2.7 Western Blot

Transfected T cells were stimulated and washed with PBS as above and lysed in Beadlyte Cell Signaling Universal Lysis Buffer (Upstate) supplemented with Halt Protease and Phosphatase Inhibitor Cocktail (Thermo Fisher). Proteins were denatured in SDS sample buffer, resolved by SDS‐PAGE using 10% Mini‐PROTEAN TGX Gel (Bio‐Rad), and transferred to Protran nitrocellulose membranes (Amersham GE Healthcare). Then membranes were blocked with 5% nonfat dry milk in TBS containing 0.1% (w/v) Tween 20 (TBST) and incubated with primary (see below) and HRP‐conjugated secondary Abs (Santa Cruz Biotechnology). Protein bands were developed with Immobilon Western Chemiluminescent HRP substrate (Millipore) in a Vilber Fusion Solo S chemiluminescence acquisition system (Vilber Lourmat). Only nonsaturated bands were quantified with ImageJ software version 1.5Oi. Abs against PPP1R11 (clone D‐9, Santa Cruz Biotechnology), PTPN22 (clone G‐3, Santa Cruz Biotechnology), NFAT1 (clone 4G6‐G5, Santa Cruz Biotechnology), phospho‐IκBα (S32/36, clone 5A5, Cell Signaling Technology), NF‐κB phospho‐p65 (S529, BD Pharmingen), NF‐κB p65 (clone D14E12, Cell Signaling Technology), MAPK p38α (clone L53F8, Cell Signaling Technology), PP1α (Atlas Antibodies), and alpha‐Tubulin (clone B‐5‐1‐2, Sigma–Aldrich) were used for Western Blot to detect corresponding targets. Where applicable, the membranes were stripped with Restore™ PLUS western blot stripping buffer (ThermoFisher Scientific) between probing steps. To avoid interference of residual bound anti‐p‐p65 Ab with binding of anti‐p65, the same lysates were run on separate gels/blots and probed separately for p‐p65 and p65, each normalized to the corresponding tubulin signal.

### 2.8 Flow cytometry

Staining with anti‐HLA‐A2‐PE (clone BB7.2, BD Biosciences), anti‐HLA‐A2‐FITC (clone BB7.2, BD Biosciences), anti‐CD4‐PerCP (clone SK3, BD Biosciences), anti‐CD4‐PE (clone #11830, R&D systems), anti‐CD25‐PE (clone 4E3, Miltenyi Biotec), and/or anti‐CD3‐PE‐Vio770 (clone BW264/56, Miltenyi Biotec) was performed in the dark with Ab diluted in FACS buffer (PBS with 0.5% HSA) for 15 min at 20°C or 30 min at 4°C. Cells were washed once with PBS, resuspended, and measured in FACS buffer. Where noted, following surface staining, viability staining was performed with Fixable Viability Dye eFluor 780 (eBioscience) according to the manufacturer's instruction (without subsequent fixation). Live cells were gated via fsc/ssc and, where applicable, on viability dye‐negative cells. Backgating of viability dye positive and negative populations confirmed that dead cells could be completely gated out by fsc/ssc with the purified CD4 T cells used. Acquisition was performed on a CyAn ADP 9 Color Analyzer (Beckman Coulter) or BD FACSVerse (BD Biosciences), and automatic parameter compensation was performed automatically with the CyAn software (Summit) tool or with FlowJo utilizing single stained control samples. Flow cytometry data were analyzed using FlowJo software (Tree Star) version 10.4.1.

### 2.9 CFSE‐based proliferation assay

For proliferation assays T cells were labeled with 2.5 µM carboxyfluorescein succinimidyl ester (CFSE; Molecular Probes via Thermo Fisher Scientific) for 8 min. The staining was stopped with PBS containing 33.34% (v/v) human serum (5 min incubation in dark while rotating) before washing with X‐VIVO 15 medium supplemented with serum. The cells were finally taken and rested overnight in X‐VIVO 15 medium (without serum). The rested cells were washed with PBS and treated with siRNAs as described earlier for nucleofection. The siRNA‐treated cells were stimulated for 4 to 5 days with plate‐bound anti‐CD3 and soluble anti‐CD28 Abs as described earlier for T cell activation prior to measurement of proliferation by CFSE‐based flow cytometry, or analysis of cytokines in the supernatant by bead‐array immunoassay.

### 2.10 Tautomycetin treatment and cytokine measurement by bead‐array immunoassay

A total of 400,000 T cells per well (96 U plate) were pre‐incubated with 50–300 nM tautomycetin (Tocris) for 5 h at 37˚C and subsequently 200,000 cells per well (96 U plate) were processed for RNA analysis after 3 h of soluble cross‐linked anti‐CD3/anti‐CD28 Ab stimulation and 5.5 days plate‐bound anti‐CD3 and soluble anti‐CD28 Ab stimulation as described above. Culture supernatants were frozen for cytokine analysis with multiplex bead‐array immunoassay after 5.5 days of stimulation. Alternatively, supernatants from siRNA‐treated cells, stimulated for 4 to 5 days as described in Section 2.4, were used for multiplex assay.

The multiplex bead‐array immunoassay was performed as previously described.^31^ All monoclonal capture Abs, biotinylated polyclonal detection Abs, and human recombinant cytokine standards were purchased from R&D systems. The capture Abs were coupled to individual magnetic carboxylated bead sets (Luminex) according to the manufacturer's recommendations. Standard curves were generated by resuspending human recombinant cytokines at concentrations ranging from 5 to 10 ng/ml and diluted serially 1:3. Assay procedures were performed in a buffer containing PBS with 0.1% BSA. The assay was run using 2000 beads per bead set in a total sample volume of 50 µl per well. T cell supernatants were run in technical unicates or duplicates. Fifty microliters of suitably diluted samples was added to the bead mixture and incubated overnight at 4˚C in a Bio‐Plex Pro flat bottom 96‐well plate (Bio‐Rad). Plates were washed twice with PBS containing 0.05% Tween 20. Biotinylated detection Abs were used at previously optimized concentrations and mixed with beads for 1 h at room temperature. Plates were washed twice, and the beads were incubated with PE‐conjugated streptavidin solution (6 µg/ml; ThermoFisher Scientific) for 30 min at room temperature. Beads were acquired with a Bio‐Plex200 system (Bio‐Rad).

The median fluorescence intensity of 100 beads per each bead set was recorded in each sample and analyzed with the Bio‐Plex Manager software 6.1 (Bio‐Rad) using a 5P regression algorithm.

### 2.11 Transcriptomic analysis of siRNA‐treated samples by RNAseq

T cells were treated with siRNA as described above. Cells were stimulated with cross‐linked anti‐CD3 and anti‐CD28 Abs for 6 h except the unstimulated samples. To extract total RNA for the RNAseq experiment, fresh cell pellets were washed twice with PBS and lysed in RLT buffer (Qiagen) supplemented with 142 mM β‐mercaptoethanol (Sigma–Aldrich), and homogenized using Qia Shredder columns (Qiagen). Lysates were frozen on dry ice and stored at –80°C until processing. RNA was extracted with the AllPrep RNA/DNA Mini Kit (Qiagen) according to the manufacturer's recommendations with minor modifications as described below. RNA was eluted with 40 µl of RNase‐free water and elution was repeated with the eluate from the first elution. RNA quality was determined with an Agilent RNA 6000 Pico Kit on a 2100 Bioanalyzer instrument (Agilent Technologies) and RNA integrity numbers (7–9.5). Randomization of samples was done to ensure that donor, time point of stimulation, and control or PPP1R11‐silenced samples were discontinuous to ensure optimal separation of biological and technical variability. Library preparation for sequencing was done in single batch. Sequencing of libraries was done in 3 batches with 9 to 10 libraries per lane. Sequencing libraries were prepared from 500 ng of total RNA using the Illumina TruSeq mRNA Stranded Library Preparation Kit (cat. no. RS‐122‐2103) according to the manufacturer's protocol (Illumina). Quality and quantity of the libraries were determined using the Agilent High Sensitivity DNA Kit (cat. no. 5067‐4626) on a 2100 Bioanalyzer instrument (Agilent Technologies) Quantification for samples for sequencing was done using the Qubit dsDNA HS Assay Kit (Cat. No. Q32854, Thermo Fisher Scientific). Sequencing of libraries was carried out on the Illumina HiSeq 2500 as per the manufacturer's instructions. ∼20 × 10^6^, 100 bp paired‐end reads were obtained per sample. Illumina adapters were trimmed from the reads using Cutadapt v1.9.1^32^ and quality of the reads was assessed using FastQC v0.11.4,^33^ followed by alignment to human genome (Ensembl GRCh 37) using TopHat2 v2.1.1.^34^ Reads were counted by using Ht‐seq in genes using the parameters (–m union –s reverse).^35^ Genes with greater than 1 read per million in more than 3 samples were included in the analysis, leaving 11,879 genes (Ensembl gene IDs) for further analysis.

Data were normalized for gene length, GC content, and library size using CQN^36^ and batch correction for batch of sequencing run with COMBAT.^37^ Here, count data was transformed to Log_2_ and transformed count data was used in the following steps. Differential expression analysis was carried out using the LIMMA package in R for the following contrasts: (i) (PPP1R11 siRNA 0h – control siRNA 0h) and (PPP1R11 siRNA 6h – control siRNA 6h) to extract differences upon siRNA treatment for each time point individually, and (ii) ((PPP1R11 siRNA 6h – PPP1R11 siRNA 0h) – (control siRNA 6h – control siRNA 0h)) to extract the interaction term representing the differential effect of siRNA treatments in shaping the response to TCR stimulation. “0h” represents unstimulated cells, and “6h” represents cells stimulated for 6 h with cross‐linked anti‐CD3 and anti‐CD28 Abs.

The linear model used for differential expression considered the following explanatory variables: donor, stimulation time, and siRNA treatment. Results were considered significant if *P* < 0.05.

Classification into lowly expressed genes (LEGs) and highly expressed genes (HEGs) was performed as follows. A total of 11,879 genes expressed above the minimal count cutoff defined above were considered in the analysis. We first confirmed that the genes in our data set were expressed in a bimodal fashion, representing the groups of LEGs and HEGs as described.^38^ We then fitted on the log_2_(normalized count) values a 1‐dimensional normal mixture model with 2 components and variable variance with mclust,^39^ considering the mean log_2_(normalized count) values of all samples. A threshold of 0.38 (for log_2_(normalized count), mean of all samples) was defined to distinguish genes classified as HEG or LEG respectively.

Gene ontology (GO) analysis by process network and pathway maps was conducted in Metacore software according to software provider's instructions.

Raw data from RNAseq have been deposited in the Gene Expression Omnibus (GEO) data repository under the accession number: GSE124757.

### 2.12 Statistical analysis

Statistical analyses for RNAseq experiment were performed in R programming language (see above). Other statistical analyses were performed in GraphPad Prism Version 7. Statistical tests used are noted in individual figure legends and/or individual parts in the Materials and Methods section.

## 3 RESULTS

### 3.1 Tregs lower the activation‐induced phosphorylation of PPP1R11 in conventional T cells

**Figure 1:**
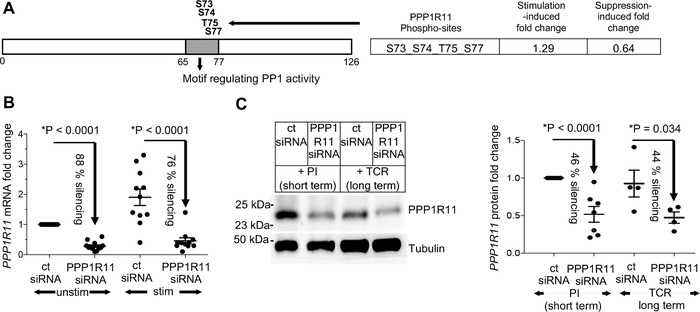
**siRNA‐mediated PPP1R11 silencing in T cells**. (**A**) PPP1R11 phosphopeptides with activation‐induced phosphorylation that is dampened by Tregs included most of the known phosphosites (4 out of 5) in the PPP1R11 motif crucial for regulating PP1 activity (grey). (**B** and **C**) T cells were treated with 2 µM of PPP1R11 siRNA pool or nontargeting siRNA as control for 4.5 days at 37˚C. The cells were then stimulated for 3 h (mRNA studies) or 4.5 days (long‐term protein studies) with cross‐linked anti‐CD3/‐CD28 (TCR) stimulation or for 5 to 30 min (short‐term protein studies) with P/I stimulation at 37˚C. (**B**) Respective mRNA levels, measured by qRT‐PCR were normalized to GAPDH and represented as fold changes compared to expression levels in unstimulated cells treated with control siRNA (set to 1). Efficiency of PPP1R11 silencing on mRNA level is shown from 12 donors (*P* < 0.0001) (mean ± sem). (**C**) Silencing efficiency of PPP1R11 on protein level was measured by quantifying Western blots and normalizing to tubulin protein. Blot displays a representative donor while figure on the right represents averaged values represented as fold change compared to expression level upon control siRNA treatment with PI stimulation (set to 1). *P* < 0.0001 for PI stimulation, *N* = 8 donors, and *P* = 0.034 for TCR stimulation, *N* = 4 donors; mean ± sem. Additionally, percentages of PPP1R11 silencing as compared to control siRNA treatment are also denoted individually for both short and long term stimulation. (**B and C**) *P*‐values were determined by one sample, unpaired 2‐sided *t*‐test (^*^ *P* < 0.05) except for stimulated samples in (B) and long‐term TCR‐stimulated samples in (C) where paired 2‐sided Student's *t*‐test was used

In order to understand the Treg‐induced changes in the TCR signaling cascade in T cells, we have previously performed global phosphoproteomic analysis of T cells upon TCR stimulation and suppression by Tregs. We observed enrichment of phosphatases among the phosphoproteins that were differentially phosphorylated both upon TCR stimulation and Treg induced‐suppression. Hence, we investigated the phosphoproteomic data for phosphatases and their known regulators. We identified phosphatase inhibitor PPP1R11 to be phosphorylated upon TCR stimulation and conversely dephosphorylated upon Treg‐mediated suppression.^10^ The differentially phosphorylated PPP1R11 phosphosites, Ser73, Ser74, Thr75, and Ser77, are located in the motif spanning across amino acids 65 and 77 of PPP1R11 (Fig. 1A) and include most of the known phosphosites (4 out of 5) in this PPP1R11 motif. Notably, this motif has been shown to be crucial to regulate the activity of the target phosphatase PP1.^40^ Differential phosphorylation of these crucial residues upon TCR stimulation and Treg‐mediated suppression (Fig. 1A) suggests that PPP1R11 may mediate both TCR‐induced stimulation and suppression of T cells via Tregs. Hence, we hypothesized that PPP1R11 could be a key molecule mediating the suppression of T cells by Tregs.

### 3.2 PPP1R11 knockdown in primary T cells by siRNA

In order to explore the possible biological role of PPP1R11 in T cell biology, we first optimized siRNA‐mediated knockdown of PPP1R11 using a pool of anti‐PPP1R11 siRNAs by Amaxa nucleofection. Dose titration of siRNA was performed using 1 and 2 µM of PPP1R11 siRNA pool. Two micromolar of siRNA achieved higher knockdown of PPP1R11 than 1 µM (mean silencing efficiency 62% on mRNA level after 3 days of incubation with siRNA and 31% of protein knockdown after 4 days of incubation, as compared to the expression level of PPP1R11 with nontargeting control siRNA (ct siRNA)) (Supplementary Fig. 1A and B). Using 2 µM of siRNA and incubation time of 4.5 days with siRNA, we achieved 76–88% of average knockdown on mRNA level in unstimulated and 5 min TCR‐stimulated cells, respectively, (Fig. 1B) and around 45% of average knockdown on PPP1R11 protein level (Fig. 1C). Notably, knockdown of PPP1R11 protein after 4.5 days incubation with siRNA was stable not only in short‐term stimulated T cells (5–30 min of PMA and Iono stimulation), but even persisted when T cells were stimulated for additional 4.5 days with TCR stimulation (Fig. 1C). Importantly, although nucleofection for transfection with siRNAs generally decreased viability, PPP1R11 silencing (as compared to control siRNA treatment) did not affect viability in T cells (Supplementary Fig. 1C).

### 3.3 Loss of PPP1R11 renders T cells partially resistant to Treg‐mediated suppression of cytokine expression

**Figure 2:**
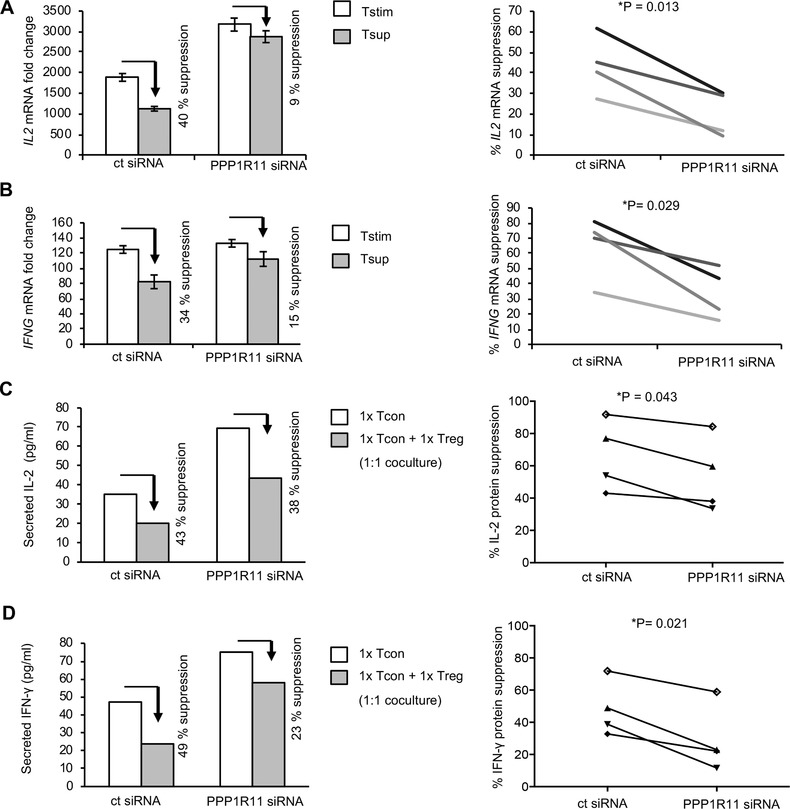
**siRNA‐mediated PPP1R11 silencing renders T cells resistant to Treg‐mediated suppression**. T cells were treated with 2 µM of PPP1R11 siRNA pool or nontargeting siRNA as control for 4.5 days. (**A and B**) Responder T cells were then cocultured with allogenic Tregs or control T cells as effectors for 75 min at 37˚C and stimulated in respective cocultures for 3 h with cross‐linked anti‐CD3/‐CD28 (TCR) Abs. Individual mRNA levels were measured in responder T cells (Tstim and Tsup) post coculture separation by qRT‐PCR. The mRNA levels were normalized to GAPDH and are represented as fold changes compared to expression levels in unstimulated cells treated with control siRNA (set to 1). Figures on the left side are representative donors of respective cytokine expressions for 4 donors (mean ± sd of PCR duplicates). Percentage of Treg‐mediated suppression of cytokine mRNA expression in suppressed cells (Tsup, grey) after removal of Tregs is additionally shown in comparison to respective cytokine expression in control cells (Tstim, white) after removal of allogenic T cells for both control and PPP1R11 siRNA‐treated T cells. Right: Individual values depict percentage of suppression of individual mRNAs between Tstim and Tsup upon treatment with respective siRNAs (*P* = 0.013 for *IL2* and *P* = 0.029 for *IFNG)* represented by different colored line per donor. *P*‐values were determined by paired, 2‐sided Student's *t*‐test (^*^ *P* < 0.05). (**C and D**) Responder T cells after treatment with respective siRNAs were either stimulated alone, or in 1:1 cocultures with Tregs for 4.5 days at 37˚C with plate‐bound anti‐CD3 and soluble anti‐CD28 Ab stimulation. Respective cytokine concentrations in the supernatant were measured by multiplex bead‐array immunoassay and concentrations are represented by figures on the left (representative of 4 donors). Percentage of Treg‐mediated suppression of secreted cytokines in supernatants from respective Tcell:Treg coculture (grey) is additionally shown in comparison to respective cytokine concentration from T cells alone (white) for both control and PPP1R11 siRNA‐treated T cells after stimulation. Right: individual values depict percentage of suppression of individual cytokines from supernatants (between the samples with and without Tregs) after treatment with respective siRNAs (*P* = 0.043 for IL‐2 and *P* = 0.021 for IFN‐γ) represented by different symbols per donor. *P*‐values were determined by paired, 2‐sided Student's *t*‐test (^*^ *P* < 0.05)

We have previously established that Tregs can rapidly suppress expression of T cell stimulation‐induced cytokines like *IL2* and *IFNG* in target conventional T cells (Tcons) upon 3 h of TCR stimulation.^41^ We used this established allogeneic Tcon:Treg coculture setting to study the effect of PPP1R11 silencing on modulating the response of T cells toward Treg‐mediated suppression. PPP1R11‐silenced T cells and control siRNA‐treated cells were cocultured with HLA‐A2‐disparate effector Tregs or effector Tcon (control). We measured the resulting *IL2* and *IFNG* cytokine mRNA in PPP1R11 siRNA‐treated target T cells post coculture separation and used it to assess the activation status of these T cells. While we observed Treg‐mediated suppression of these cytokines in control siRNA‐treated cells, the extent of Treg‐mediated suppression was significantly reduced in PPP1R11 siRNA‐treated cells (*P* = 0.013 for *IL2* and *P* = 0.029 for *IFNG* mRNA; Fig. 2A and B). Additionally, we measured secreted cytokine protein concentrations in the supernatants from Tcon:Treg cocultures following 4.5 days of activation. Similar to the cytokine mRNA studies, we observed resistance toward Treg‐induced suppression of IL‐2 and IFN‐γ cytokines in the supernatants upon PPP1R11 siRNA treatment (*P* = 0.043 for IL‐2 and *P* = 0.021 for IFN‐γ; Fig. 2C and D). Tcon cultures without Tregs were taken as positive control (set to 100% for calculating percent suppression by Tregs), while cultures comprising only Tregs were taken as negative controls. Cytokine concentrations in the negative controls were below detection limit (data not shown), confirming that Treg produced cytokines did not affect the coculture readings and in line with the well‐known anergy of Tregs in vitro with respect to expression of these cytokines. This loss of susceptibility of T cells toward suppression by Tregs upon PPP1R11 silencing suggests that loss of PPP1R11 induces resistance in T cells toward Treg‐mediated suppression of TCR activation‐induced cytokines.

### 3.4 PPP1R11 silencing efficiency reflects the observed resistance to Treg‐mediated suppression of T cells

In order to negate possible off‐target effects resulting from the use of siRNA pools, we verified the biological effect of PPP1R11 using individual PPP1R11 siRNAs chosen from the siRNA pool. Four individual PPP1R11 siRNAs were screened for their silencing efficiency on T cells using the same conditions and dosages as for the PPP1R11 siRNA pool. Two out of four siRNAs represented as PPP1R11‐07 and PPP1R11‐08 were selected for further experiments due to their superior PPP1R11 silencing efficiency (79 and 60%, respectively) in unstimulated T cells (Supplementary Fig. 1D). Next, we subjected T cells, post PPP1R11 silencing with these 2 individual PPP1R11 siRNAs along with the previously used PPP1R11 siRNA pool, to Treg‐mediated suppression as described above. As observed earlier, individual PPP1R11 siRNAs also induced resistance toward Treg‐mediated suppression in T cells (74 and 32% abrogation of *IL2* suppression with PPP1R11‐07 and PPP1R11‐08, respectively).

More interestingly, the abrogation of *IL2* mRNA suppression by individual PPP1R11 siRNAs and pool were proportional and correlated to their respective PPP1R11 silencing efficiency (Pearson correlation coefficient = 0.99; Supplementary Fig. 1E). This serves as an indication that PPP1R11 silencing is causative for resistance of T cells toward Treg‐mediated cytokine suppression.

### 3.5 PPP1R11 silencing imparts an activated phenotype to T cells, leading to increased cytokine secretion

**Figure 3:**
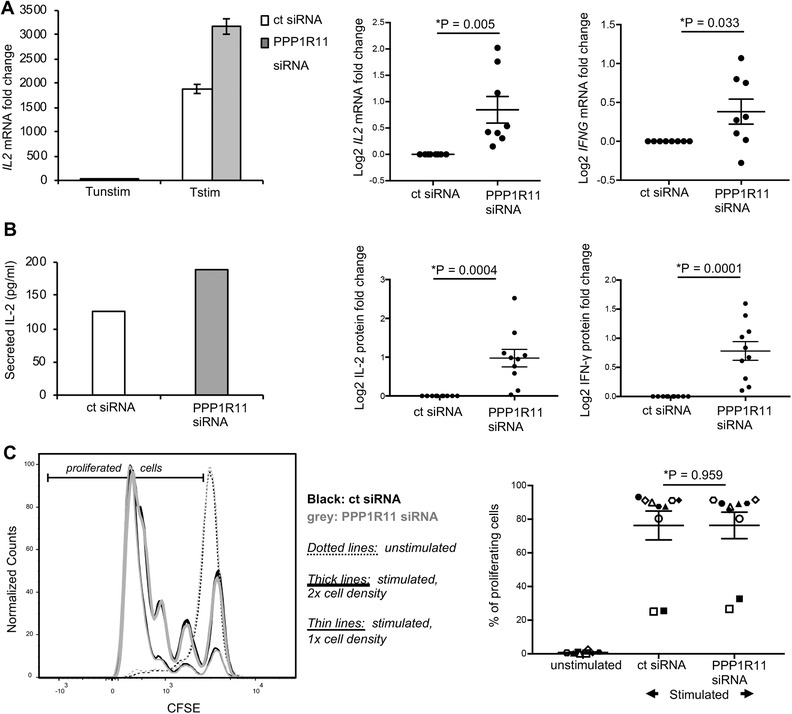
**PPP1R11 silencing augments TCR‐induced cytokine expression in T cells**. T cells were treated with 2 µM of PPP1R11 siRNA pool or nontargeting siRNA for 4.5 days and then activated for (A) 3 h (mRNA studies) with cross‐linked anti‐CD3/‐CD28 (TCR) Ab stimulation or (B and C) 4.5 days with plate‐bound anti‐CD3 and soluble anti‐CD28 Abs at 37˚C. (**A**) Respective cytokine mRNA levels measured by qRT‐PCR were normalized to *GAPDH* and represented as fold changes compared to expression levels in unstimulated cells (set to 1) treated with respective siRNAs. (Left) Representative figure for *IL2* mRNA (mean ± sd of PCR triplicates) expression upon treatment with control siRNA (white bars) and PPP1R11 siRNA (grey bars). (Middle and right) Averaged log_2_ value for respective mRNAs (mean ± sem of 8 donors) (*P* = 0.005 for *IL2* and *P* = 0.003 for *IFNG*) are expressed as fold change with respect to control siRNA (log_2_ set to 0). *P*‐values were determined by unpaired, one sample, 2‐sided *t*‐test (^*^ *P* < 0.05). (**B and C**) T cells were labeled with CFSE prior to siRNA‐treatment. (**B**) Respective cytokine concentrations in the supernatants of T cells treated with respective siRNAs were measured by multiplex bead‐array immunoassay and represented by figures on the left. (Middle and right) Averaged figures for respective cytokine concentrations (mean ± sem of 10 donors; *P* = 0.0004 for IL‐2 and *P* = 0.0001 for IFN‐γ) are expressed as fold change with respect to control siRNA‐treated cells (log_2_ set to 0). *P*‐values were determined by unpaired, one sample, 2‐sided *t*‐test (^*^ *P* < 0.05). (**C**) Left panel: Proliferation was assessed by flow cytometry by gating on live (viability dye‐negative), singlet, CD3^+^CD4^+^CFSE^+^ T cells. Unstimulated T cells (dotted lines) were used as control. Overlaid CFSE histograms are shown for a representative donor (for control siRNA‐treated cells in black and PPP1R11 siRNA‐treated cells in grey, and for 2 different starting cell densities each as indicated by thick or thin lines, respectively). The right panel represents averaged values for percentage of proliferating cells (gate as indicated in the left panel) from 10 donors in 3 separate experiments (mean ± sem). Each symbol represents an individual donor. Percentage of proliferating cells was calculated as percentage of CFSE‐diluting cells of total CFSE‐positive cells. *P*‐values were determined by paired, 2‐sided Student's *t*‐test

To understand the cause of apparent resistance toward Treg‐mediated suppression upon PPP1R11 silencing, we next dissected the direct effect of PPP1R11 silencing on expression of various T cell activation‐induced cytokines independent of Tregs. We observed significantly up‐regulated expression of the cytokines *IL2* (*P* = 0.005) and *IFNG* mRNA (*P* = 0.033) in T cells treated with PPP1R11 siRNA pool as compared to control siRNA‐treated cells after 3 h of TCR stimulation (Fig. 3A). We further confirmed the specificity of this effect by de‐convoluting the role of individual siRNAs in the PPP1R11 siRNA pool. We analyzed cytokine expression after silencing with individual PPP1R11 siRNAs (PPP1R11‐07 and PPP1R11‐08), and PPP1R11 silencing with these individual siRNAs also up‐regulated TCR stimulation‐induced *IL2* mRNA expression as compared to control siRNA‐treated cells after 3 h of TCR stimulation (Supplementary Fig. 1F). Along with increased expression of *IL2* and *IFNG* mRNA, PPP1R11‐silenced cells also secreted higher concentrations of IL‐2 (*P* = 0.0004) and IFN‐γ (*P* = 0.0001) protein into the supernatant after 4.5 days of activation (Fig. 3B).

We further investigated whether PPP1R11 silencing affects other T cell activation‐induced molecules. We observed that PPP1R11 silencing also up‐regulated the TCR stimulation‐induced expression of *CD69* (*P* = 0.001), a marker of early T cell activation while late activation markers like *IL2RA* or *CTLA4* were not significantly affected upon PPP1R11 silencing (Supplementary Fig. 2).

### 3.6 Mechanistic aspects of cytokine upregulation in PPP1R11‐silenced T cells

Our data suggests that PPP1R11‐silenced cells respond differentially to TCR stimulation compared to control siRNA‐treated T cells. Hence, PPP1R11 silencing may affect intracellular signaling of T cells downstream of the TCR. First, we checked whether general responsiveness to TCR stimulation may be affected due to reduced levels of the TCR complex on the surface. Hence, we exemplarily measured surface levels of CD3ε, which were not altered in PPP1R11 silenced T cells (Supplementary Fig. 3A). To further discern the position of PPP1R11 in the TCR signaling cascade, we stimulated the PPP1R11‐silenced cells with a combination of PMA and Iono (P/I), which are a diacylglycerol analogue and Ca^2+^ ionophore, respectively, and which affect an intermediate segment of the TCR signaling cascade. We found that PPP1R11‐silenced cells, compared to control siRNA‐treated cells, also presented with increased expression of *IL2* and *IFNG* mRNA in response to P/I stimulation, similar to the earlier observation with TCR stimulation (Supplementary Fig. 3B). Differential up‐regulation of these T cell stimulation‐induced cytokines with P/I stimulation indicates that PPP1R11 affects an intermediate stage of TCR signaling where targets of P/I stimulation lie. However, this does not exclude that T cell signaling at the TCR‐proximal stage or unknown pathways independent of classical TCR signaling may be affected in addition. Three major pathways downstream of the TCR lead to activation of key transcription factors in T cells (AP‐1, NFAT, and NF‐κB) that contribute to cytokine gene expression, and TCR signaling is largely mediated by posttranslational modifications such as phosphorylation.^42^ Since PMA activates PKCθ and Ras affecting NF‐κB and MAPK pathways, and Iono activates NFAT and augments NF‐κB signaling, we assessed PPP1R11‐silenced cells for alterations of classical molecules downstream of PMA/Iono in these pathways. However, we did not observe significant effects on the phosphorylation or total levels of exemplary canonical signaling molecules in NFAT, NF‐κB, and MAPK pathways upon PPP1R11 silencing following PMA/Iono or TCR stimulation (Supplementary Fig. 3C).

Taken together, PPP1R11 silencing‐induced up‐regulation of *IL2* and *IFNG* indicates PPP1R11 as a novel negative regulator of T cell activation‐induced cytokine expression not affecting the here assessed molecules in classical TCR signaling pathways.

### 3.7 PPP1R11 silencing augments T cell cytokine expression without affecting proliferation

TCR plus co‐stimulation does not only lead to cytokine expression, but also to proliferation of T cells. At the same time, cytokine expression can be modulated independently of affecting proliferation, as distinct TCR signaling pathways drive proliferation and cytokine production,^12^ and proliferation can still occur if major TCR signaling pathways and consequently cytokine expression are disturbed.^43^ Along these lines, interestingly, Tregs also seem to utilize different and partially independent mechanisms to suppress cytokine expression and proliferation in responder T cells.^13^, ^42^, ^44^ Cytokine suppression seems to be most relevant for direct suppression of T cells by direct contact to Tregs as studied here and shown to occur in vivo in immune effector phases in nonlymphoid target tissue.^45^ On the other hand, inhibition of proliferation seems to be mostly a result of indirect suppression of T cells by Tregs via contact between Tregs and APCs,^46^, ^47^, ^48^ which also occurs in vivo in lymphoid tissue.^47^, ^49^, ^50^ Complicating the situation, secreted cytokines, especially IL‐2, feedback to enhance T cell proliferation.^51^ Therefore, to examine the potential effect of PPP1R11‐silencing induced upregulation of cytokines such as IL‐2 on proliferation, or direct effects of PPP1R11 on proliferation, we compared long‐term TCR stimulation‐induced proliferation of T cells treated with PPP1R11 siRNA or control siRNA. We performed CFSE‐based proliferation assays with PPP1R11‐silenced cells upon 4 days of plate bound anti‐CD3 and soluble anti‐CD28 Abs stimulation. Stimulation‐induced proliferation of PPP1R11 siRNA‐treated cells did not show any significant difference to that of control siRNA‐treated cells (Fig. 3C).Notably and as described above, PPP1R11 protein knockdown (Fig. 1C), high viability (Supplementary Fig. 1C), and increased IL‐2 protein secretion (Fig. 2C) were observed in these long‐term stimulated cells, serving as an important control and confirming stability of knockdown effects at the late time points required to study proliferation.

The observation that PPP1R11 does not affect proliferation, taken together with our earlier observation that PPP1R11 silencing up‐regulates T cell stimulation‐induced cytokines, suggests a proliferation‐independent activation of T cells upon PPP1R11 silencing.

### 3.8 PPP1R11 silencing globally alters the activation‐induced transcriptome of T cells

To further study potential mechanisms of PPP1R11 silencing on T cell activation and building on our finding that PPP1R11 affects expression of specific T cell activation‐induced cytokines, we assessed the effects of PPP1R11 silencing on the global transcriptomic profile of T cells and the response of T cells to TCR stimulation. To this end, we performed RNAseq with T cells treated with either control or PPP1R11 siRNA for 4.5 days and stimulated with TCR stimulation for 6 h or processed in unstimulated state in 3 donors. The expression profile of identified genes clustered according to treatment (type of siRNAs and time point of stimulation; Supplementary Fig. 4), suggesting a specific effect of PPP1R11 silencing on the T cell transcriptome.

**Figure 4:**
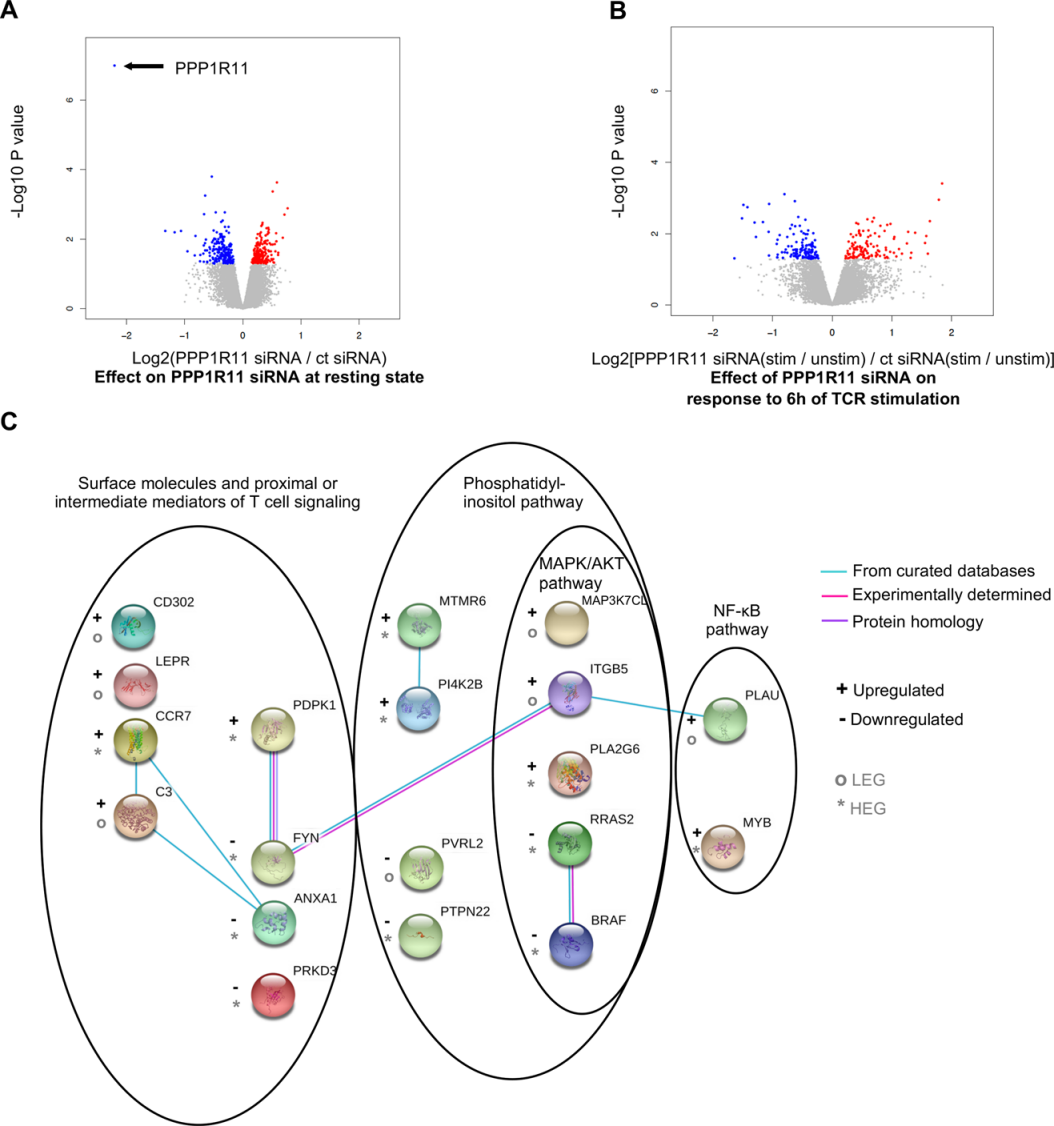
**PPP1R11 affects the baseline and T cell activation‐induced transcriptome of T cells**. T cells were treated with either control or PPP1R11 siRNA for 4.5 days and then either stimulated with cross‐linked anti‐CD3/‐CD28 Abs for 6 h or left unstimulated for RNAseq analysis. (**A and B**) For relative fold change, log_2_ of average ratios of normalized gene counts in each comparison were plotted against *P*‐value of the respective change. Differentially expressed genes (DEGs; *P* < 0.05) are colored in red (up‐regulated) or blue (down‐regulated) in each comparison. *P*‐values were calculated with moderated *t*‐test using the Limma package in R. (A) Figure represents differential effect of PPP1R11 siRNA over control siRNA at resting stage. (B) Figure represents differential response of PPP1R11 silenced cells over control siRNA silenced cells to TCR stimulation. (**C**) PPP1R11 silencing differentially regulates genes associated with phosphatidyl inositol, MAPK, AKT, and/or NF‐κB pathways upon TCR stimulation. DEGs associated with these pathways were used to curate a gene network in the STRING web‐platform. Only known interactions were used to represent connections between the nodes (color coding, see in the figure). Schematic crystal structures of the proteins are also provided where available. The nodes are roughly arranged according to the subcellular locations and genes involved in respective pathways are encircled for better visualization. “+” or “–” signs are used to represent up‐regulation or down‐regulation of genes, respectively, upon PPP1R11 silencing over control siRNA treatment. Furthermore, “*” or “°” signs are used to represent highly or lowly expressed genes (HEG or LEG), respectively

First, as a measure of stimulation‐independent effects of PPP1R11 silencing on the T cell transcriptome, we studied the genes that were differentially regulated by PPP1R11 silencing compared to control siRNA treatment at resting stage. Out of 11,879 genes that were expressed according to the applied minimal count filtering threshold (see section Materials and Methods), PPP1R11 silencing differentially regulated 417 genes (*P* < 0.05; Fig. 4A and Supplementary Table 1). *PPP1R11* itself was the most down‐regulated gene, which confirms the efficiency of our PPP1R11 silencing experiment and analysis pipeline. Similarly, we also found 2 other regulatory subunits of protein phosphatase 1 (*PPP1R12A* and *PPP1R3D*) significantly down‐regulated upon treatment with PPP1R11 siRNA in addition to *PPP1R11* itself. It is most likely that this resulted from a secondary effect of PPP1R11 knockdown and not from direct effects of the siRNAs, since the used PPP1R11 siRNA oligos do not have sequence similarity with either *PPP1R12A* or *PPP1R3D*. Indirect regulatory effects are expected, as the subunits of the PP1 holoenzyme are also well known to extensively regulate each other.^23^ Nevertheless, the extent of *PPP1R12A* and *PPP1R3D* down‐regulation upon treatment with PPP1R11 siRNA was about 15 times less than down‐regulation of PPP1R11 itself. Interestingly, PPP1R11 silencing did not cause any significant change on the mRNA level of its most well characterized target PP1A (gene name *PPP1CA*).

In order to understand which pathways are affected upon PPP1R11 silencing, we performed GO analysis with these differentially expressed genes (DEGs). The majority of the processes enriched upon PPP1R11 silencing over control siRNA treatment involved nucleic acid and cholesterol‐modulating processes (Supplementary Fig. 5A).

Next, we focused on the genes whose stimulation‐induced expression was differentially regulated by PPP1R11 siRNA treatment as compared to control siRNA treatment (also referred to as interaction term). PPP1R11 silencing differentially regulated the TCR stimulation‐induced expression of 249 genes (*P* < 0.05; Fig. 4B and Supplementary Table 1). GO analysis of these DEGs revealed involvement of immunological processes such as complement system, generation of memory T cells, PI3K‐dependent leptin signaling, and other pathways such as apoptosis and cell cycle, which are associated with T cell activation (Supplementary Fig. 5B). Building on this indication that T cell activation‐related genes are differentially regulated upon TCR stimulation of PPP1R11‐silenced cells, we manually curated a network of the DEGs that have been shown to be involved in T cell activation by GO analysis, KEGG pathway,^52^ PathCards,^53^ and literature review in the STRING web‐platform.^54^ DEGs appearing in this network included surface molecules or mediators of T cell signaling such as C‐C motif chemokine receptor 7 (*CCR7*), *FYN* kinase, leptin receptor (*LEPR*), among others. Notably, we also observed multiple molecules associated with either or both phosphatidylinositol pathway and AKT/MAPK pathway such as 3‐phosphoinositide dependent protein kinase 1 (*PDPK1*), phosphatidylinositol 4‐kinase type 2 beta (*PI4K2B*), related RAS viral (r‐ras) oncogene homolog 2 (*RRAS2*), v‐raf murine sarcoma viral oncogene homolog B (*BRAF*), integrin beta 5 (*ITGB5*), and MAP3K7 C‐terminal like (*MAP3K7CL*) (Fig. 4C). We also found molecules associated as downstream products of the NF‐κB pathway such as plasminogen activator, urokinase (*PLAU*), and v‐myb avian myeloblastosis viral oncogene homolog (*MYB*) (Fig. 4C). Taken together, although above inspection of common TCR signaling phosphoproteins did not reveal alterations in the upstream pathways (Supplementary Fig. 3), our data suggest that the PPP1R11 silencing‐induced effect on T cell activation‐induced cytokines might affect MAPK, AKT, and/or NF‐κB signaling pathways at least on the level of RNA expression.

### 3.9 Validation of the RNAseq study on protein level

While RNAseq data and pathway analyses can give indications about processes involved in transcriptional regulation of DEGs, it needs to be considered that not all DEGs are necessarily also regulated on protein level or even expressed^55^ (even when considering only protein‐coding genes). Indeed, protein and RNA expression can be concordant for certain genes, but at the same time discordant for others, as shown for many cell types and specifically for primary human T cells in parallel RNAseq and proteomics studies.^56^, ^57^ An additional complication is the potentially different dynamics of RNA and protein expression, which can preclude protein level validation if the time points chosen for validation are suboptimal. One way to classify transcripts that are more likely to have a functional protein product is by their relatively high expression level, as it was shown that RNAseq reveals two major classes of gene expression levels, so‐called HEGs and LEGs.^38^ Although HEGs are more often detected on proteome level than LEGs, high expression of a protein already in the steady state (and hence, strong signals when detecting the respective protein) can make it more difficult to detect subtle changes upon treatment. We classified the genes analyzed in this RNAseq study (after the expression threshold filtering rule applied above that considers only genes with greater than one read per million in more than 3 samples) and revealed that ∼86% of all detected genes were HEGs (Supplementary Fig. 6A). Within the DEGs studied above (Fig. 4A and B), a similar fraction (88%) belonged to the class of HEGs (Supplementary Fig. 6B). Based on these criteria focusing on HEGs, together with biological relevance in T cells and availability of Abs, we chose Protein Tyrosine Phosphatase, Non‐Receptor Type 22 (PTPN22)^58^ as a suitable interesting candidate gene for validation on protein level. Indeed, PTPN22 mRNA that was down‐regulated upon PPP1R11 knockdown in TCR‐stimulated cells in RNAseq data was confirmed to be down‐regulated on protein level in independent donors (Supplementary Fig. 6C, E, and F). Another DEG candidate for protein validation, LEPR, was not detectable on protein level in the cells under study (data not shown), in accordance with its classification as LEG. Further, PPP1R11 itself, the most strongly down‐regulated DEG in our RNAseq analysis and classified as HEG is another striking example that could be validated on protein level in independent donors as described earlier (Fig. 1). We also observed that PP1A (PPP1R11‐target), which was not transcriptionally regulated by PPP1R11 in our RNAseq analysis, was similarly not regulated on protein level either (Supplementary Fig. 6D). Notably, although the effect of PPP1R11 silencing to up‐regulate *IL2* and *IFNG* mRNA level was used as a quality control (by qRT‐PCR) prior to RNAseq analysis, our cutoffs in the RNAseq data of the corresponding samples did not reveal *IL2* and *IFNG* as DEGs (*P* > 0.05) despite a trend of up‐regulation, which was also confirmed above on the secreted protein level of IL‐2 and IFN‐γ cytokines (Fig. 3B). Together, these data confirm the validity of our RNAseq data and analysis. Nevertheless, potential follow‐up studies focusing on certain DEGs as discovered here should always include protein level verification for the specific gene of interest, as protein:RNA correlation may differ for each individual gene.

### 3.10 Inhibition of PP1 attenuates pro‐inflammatory cytokine expression in T cells

**Figure 5:**
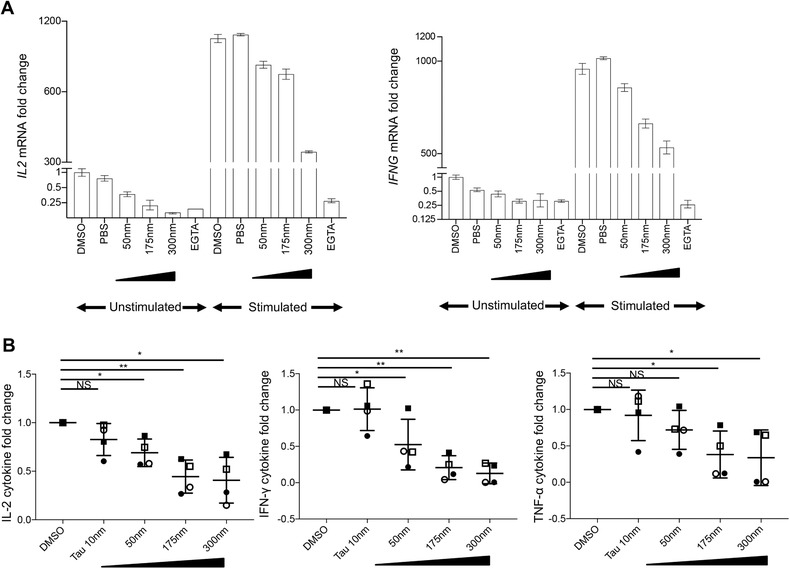
**Chemical inhibition of the PPP1R11 target PP1 dampens T cell activation‐associated cytokines**. T cells were treated with indicated concentrations of PP1 inhibitor tautomycetin for 5 h at 37˚C and activated for (**A**) 3 h with soluble cross‐linked anti‐CD3/‐CD28 Abs for mRNA studies or (**B**) 5.5 days with plate‐bound anti‐CD3 and soluble anti‐CD28 Abs for cytokine studies in the supernatant at 37˚C. DMSO‐treated and PBS‐treated cells were used as vehicle control and negative control, respectively, while EGTA‐treated cells were used as positive control for mRNA studies. DMSO‐treated cells were used as vehicle control for cytokine studies in the supernatant. Both unstimulated and stimulated cells were measured for mRNA studies by qRT‐PCR while only stimulated cells were measured for cytokine protein studies by multiplex bead‐array immunoassay. (A) Respective cytokine mRNA levels were normalized to GAPDH and are represented as fold changes compared to expression levels in unstimulated DMSO‐treated cells (set to 1). Representative results for *IL2* mRNA (left) and *IFNG* mRNA (right) from 2 donors are shown (mean ± sd of PCR triplicates). (B) Respective cytokine concentrations for varying doses of tautomycetin are represented as fold change compared to cytokine concentration for DMSO‐treated cells (set to 1). Averaged result for IL‐2 (left), IFN‐γ (middle), and TNF‐α (right) is shown (mean ± sem of 4 donors). *P*‐values were determined by paired, one‐sample, 2‐sided *t*‐test (^*^ *P* < 0.05 and ^**^ *P* < 0.005). Individual symbols represent individual donors

Taken together, our data suggests PPP1R11 to be a negative regulator for T cell activation‐induced cytokines, although it is still unknown what exactly mediates the effect of PPP1R11 on T cells. The most well‐characterized function of PPP1R11 is inhibition of the PP1 enzyme complex activity.^21^ As described above, we did not observe a significant change of PP1A mRNA or protein levels upon PPP1R11 silencing (Supplementary Table 1 and Fig. 6D). We further confirmed, using data from an independent previously published study that comprises a time‐series of human naïve CD4 T cell activation with plate‐bound anti‐CD3 and soluble anti‐CD28 Abs,^57^ that the expression pattern of PPP1R11 and PP1A were not positively correlated: while *PPP1R11* mRNA peaked within 2 h of stimulation and gradually decreased then on, *PPP1CA* expression increased with stimulation in a seemingly opposite manner (data not displayed; re‐analysis of data published in ref. 57). The reciprocal expression pattern of *PPP1R11* and *PPP1CA* provides correlative evidence that PPP1R11 expression is negatively associated with T cell activation and PP1A expression. To supplement our hypothesis that PPP1R11 affects TCR activation‐induced cytokine expression via inhibition of enzymatic activity of its target PP1A, we performed chemical inhibition of PP1 by tautomycetin^59^ in the same cell type (CD4^+^ CD25^–^ Tcon) as used in above studies for PPP1R11 silencing. We then compared the short‐term and long‐term effects of PP1A inhibition on T cell activation with those effects caused by PPP1R11 silencing. To this end, Tcons were treated with various concentrations of tautomycetin for 5 h at 37˚C and activated for 3 h at 37˚C with soluble cross‐linked anti‐CD3 and anti‐CD28 Abs for RNA studies (Fig. 5A) or 5.5 days with plate‐bound anti‐CD3 and soluble anti‐CD28 Abs for cytokine protein secretion studies (Fig. 5B). DMSO‐treated and PBS‐treated cells were used as vehicle control. We observed a dose‐dependent inhibition of *IL2* and *IFNG* mRNA and corresponding cytokine secretion (*P* < 0.05 and *P* < 0.005) with tautomycetin treatment. Further, we observed similar suppressive effects on secretion of the pro‐inflammatory cytokine TNF‐α. We also measured the concentration of other T cell subset‐specific cytokines: IL‐17A and CCL20 also exhibited a similar trend of attenuation, although levels were low and detectable levels were only reached in 2 out of 4 donors (data not shown). Other cytokines measured (IL‐4 and IL‐10) were largely below the detection limit of multiplex bead‐array immunoassay. This seemingly reciprocal nature of the immunological effect of chemical inhibition of PP1 and siRNA‐mediated silencing of PPP1R11 correlatively suggest that the effect of PPP1R11 on T cell cytokine expression might be mediated by inhibiting the activity of PP1.

Altogether, our data reveal a novel role of PPP1R11 on the expression and Treg‐mediated suppression of cytokines in T cells. PPP1R11 exerted its function independent of proliferation and commonly studied upstream TCR signaling molecules. Mechanistically, PPP1R11 likely operates through modulation of the activity of PP1A, whose chemical inhibition reciprocally mimicked the effects of PPP1R11.

## 4 DISCUSSION

T cells resistant toward immunoregulation by Tregs pose a significant threat toward immunotherapeutic strategies targeted at modulating T cell activation either via Tregs or other means of immunoregulation. While breakdown of this resistance is desirable in the treatment of autoimmune diseases and transplantation settings, inducing such resistance may be favorable in case of cancer immunotherapy. Several cell extrinsic factors, mainly altered cytokine micro‐milieu involving expression of IL‐6,^60^ TNF‐α,^16^, ^61^ IL‐15^18^, IL‐21,^62^, ^63^ and IL‐4^64^, ^65^ have been shown in experimental and clinical settings to modulate resistance in T cells. Comparatively less is known about the cell intrinsic factors modulating resistance in T cells, which is probably because of a shortage of global studies on responder T cell signaling induced by Treg‐mediated suppression. We have previously generated data of global changes in protein phosphorylation patterns in T cells upon TCR stimulation and suppression by Tregs.^10^ This can be a valuable resource to understand T cell signaling shaped by TCR stimulation and Treg‐mediated suppression in general and hence further our understanding of cell intrinsic causes of resistance. Following up on the candidate molecules from our previous study, in this study, we have shown the phosphatase inhibitor PPP1R11 to mediate susceptibility of T cells toward suppression by Tregs. We are specifically studying direct (APC independent), rapid suppression of T cell‐cytokine production by Tregs. As discussed previously,^13^ this may be especially relevant to therapeutically modulate the cytokine milieu at the effector phase of inflammation in the tissue where Tregs and T cells directly interact.^45^

PPP1R11 is mainly characterized as a potent inhibitor of the PP1 phosphatase. Although the role of PPP1R11 in T cell biology is not well known, in other cell types, PPP1R11 has been shown to control crucial steps in cell cycling and apoptosis either by affecting PP1 activity or affecting its interaction with other binding partners.^26^, ^27^, ^28^ PP1 is a ubiquitous phosphatase as PP1 along with PP2 is accredited for over 90% of phosphatase activity in eukaryotic cells.^66^ The very fact that only 35 human Ser, Thr phosphatases^67^ have been identified to balance the activity of 428 Ser, Thr kinases^68^ might give an indication that protein phosphatases act in a less specific manner. However, as opposed to kinases, Ser, Thr phosphatases are heavily regulated by interacting proteins—as an example, PP1 is regulated by over 200 different PIPs acting as targeting subunits, substrates, and/or inhibitors for the catalytic core such as in the case of PPP1R11, which modulates PP1 activity.^23^ Although the catalytic core of PP1 in mammals consists of only 5 catalytic subunits: PP1α1, PP1α2, PP1β, PP1γ1, and PP1γ3 encoded by 3 genes *PPP1CA, PPP1CB*, and *PPP1CC* (also known as *PP1A, PP1B*, and *PP1C*, respectively); the PP1 holoenzyme exists as over 650 different complexes. This is mainly because these catalytic subunits uniquely combine with numerous PIPs and over 50 different regulatory subunits out of which 16 are inhibitory subunits.^69^ Existence of such diverse arrays of subunits suggests that instead of targeting the catalytic subunits like done for kinases, it may be more specific to target these regulatory subunits instead for attaining specific therapeutic outcomes. Furthermore, targeting these regulatory subunits to control cell‐ and context‐specific functions of PP1 in clinical settings has already been suggested before.^70^ To this end, our insight into a novel role of PPP1R11 in modulating T cell cytokine expression and sensitivity toward cytokine suppression by Tregs can provide interesting avenues in modulating PP1 activity specifically in T cells. PPP1R11 was shown before to specifically inhibit the biochemical activity of PP1.^21^ Here, we did not detect any significant effect of PPP1R11 on transcription or translation of PP1A. So it is likely that PPP1R11 instead regulates the substrate specificity and activity of PP1 catalytic subunit by introducing conformational changes in the holoenzyme, affecting the subcellular localization and also interacting with the PP1 substrates for competitive inhibition rather than transcriptional regulation of PP1 itself, as established in earlier studies and reviewed by Bollen and colleagues.^23^

The most striking effect of PPP1R11 silencing was up‐regulation of IL‐2 and IFN‐γ cytokines in T cells without concomitantly affecting proliferation. Although cytokine expression and proliferation in T cells often occur simultaneously when T cells are activated, they are regulated by different pathways^12^ and can be controlled independently by Tregs,^13^, ^44^ as discussed above and reviewed previously.^42^ Furthermore, we have shown before that the rapid suppression of TCR‐induced cytokine expression in T cells by Tregs occurs via mechanisms independent of APCs and cytotoxic T‐lymphocyte associated protein 4, which instead control indirect suppression of T cell proliferation.^13^, ^46^ In line with these findings, PPP1R11 affected T cell cytokine expression and suppression by Tregs without affecting proliferation. However, any negative data obtained by performing siRNA‐based silencing experiments recorded in dividing cells must be taken with caution, since siRNA concentrations decrease with each round of cell division. Yet, our data indicate that this was not necessarily a confounding factor, since even in the early cycles of division, we did not observe significant differences in proliferation for control and PPP1R11‐silenced cells, and conversely increased cytokine secretion and PPP1R11 protein knockdown were still observed at the late time points when proliferation was measured. Nevertheless, potential effects on proliferation may depend on the effect strength, i.e., whether IL‐2 levels are sufficiently reduced to observe consequently reduced proliferation or vice versa. Future studies may further address these points by using PPP1R11 knockout models.

We have previously shown that Tregs can rapidly suppress TCR‐generated cytokine expression via inhibition of NFAT and NF‐κB pathways,^13^ and found a phosphoprotein (DEF6) to confer regulation of NFAT1^10^ in this context. To decipher the mechanism, how PPP1R11 might affect TCR‐induced cytokine expression in this context, we inspected several classical TCR signaling proteins, yet we did not find alterations in the molecules studied (such as p‐p65 NF‐κB, p‐NFAT1, and p38). Strikingly, chemical inhibition of PP1—which should have the opposite effect as taking away the PP1 inhibitor PPP1R11 (by siRNA)—indeed led to reduced expression of the same cytokines (IL‐2 and IFN‐γ) in Tcons. Hence, we propose that PPP1R11 exerts its effect on T cell activation mainly through inhibiting PP1A. In agreement with this hypothesis, a report that used similar settings like ours with primary short‐term stimulated T cells, as well as different T cell lines featuring PP1A overexpression or knockdown, has reported PP1A to positively regulate the expression of IL‐2 and IFN‐γ.^24^ This report was based on a previously conducted siRNA screen in a Jurkat T cell line, which revealed PP1A (among other phosphatases) as a positive regulator of TCR‐induced NF‐κB activation.^71^ Notably, despite reduced NF‐κB reporter activity, less DNA binding, and reduced target gene expression, phosphorylations of extensively studied upstream NF‐κB signaling molecules as well as nuclear translocation of NF‐κB were basically unaffected by PP1A in T cells.^24^ This is in agreement with our findings that PPP1R11 did not alter the NF‐κB signaling molecules studied despite affecting NF‐κB target genes. Another very recently published study^25^ confirmed that PP1A affected NF‐κB signaling in T cells and consequently cytokine expression including IL‐2, IL‐10, and to a lesser extent IFN‐γ. However, it needs to be noted that the authors^25^ used PHA‐stimulated T cell blasts that were expanded in IL‐2‐containing medium before use for functional studies and costimulation, and hence the comparability to our study may be limited, while Mock et al.^24^ used basically the same primary T cell type as we do. In line with the data from Mock et al.^24^ that PP1A did not affect upstream signaling yet augmented NF‐κB activity, we determined that the PP1‐regulator PPP1R11 affected NF‐κB‐dependent cytokine expression without alterations in upstream signaling, and occurring downstream of the PMA/Iono targets, which affect an intermediate segment of TCR signaling. Despite the common scheme of NF‐κB augmentation, the exact mechanism remains elusive and may involve novel, so far unknown regulators of NF‐κB transcription factor activity, which remains a challenge to study in primary human T cells and would be an interesting subject of future investigations especially in the context of PPP1R11. The above report^24^ furthermore agrees with our finding that chemical inhibition of PP1A by tautomycetin affects T cell cytokine expression. Tautomycetin has also been shown to suppress IKK phosphorylation, and hence, signaling to NF‐κB in TNF‐stimulated non‐T cell lines.^72^ However, the use of high concentrations of tautomycetin (5 µM) and the use of non‐T cell lines^72^ render this study difficult to compare with the studies in T cells, in which despite slightly altered kinetics of IKK kinase activity no drastic effects of PP1A on IKK phosphorylation downstream of TCR as well as TNF stimulation were observed.^24^ Further, PP1A inhibition by tautomycetin was indicated to inhibit AKT phosphorylation in T cells.^73^ Although tautomycetin is a selective inhibitor of PP1 (IC50 1.6 nm) it is also known to have a weaker effect on PP2A (IC50 62 nm).^59^ However, 5 µM tautomycetin was shown to inhibit PP1A without affecting PP2A.^59^, ^72^ Hence, it cannot be excluded that depending on the cell type, the tautomycetin‐induced effect on the measured cytokines could also be in part mediated by PP2A in addition to PP1. This needs to be considered especially because PP2A itself is implicated in NF‐κB signaling in T cells.^74^ Importantly, our novel results revealing a role of PPP1R11 in TCR‐induced cytokine activation may allow for a more specific targeting of PP1A activity in T cells. In stark contrast to the role of PP1 in T cells as shown in the above literature and indicated by our study, PP1 has been shown to negatively regulate pro‐inflammatory cytokine production in macrophages by inhibiting MAPK and NF‐κB pathways.^75^ This observation may very well point at cell type‐specific effects of PP1. It is plausible that the T cell‐specific effect of PP1 is regulated by PPP1R11, similarly to the recently uncovered role of the PP2A regulatory subunit B56γ in mediating specific effects upon TCR stimulation.^74^ Parallel studies to understand the effect of PPP1R11 on multiple immune (and other) cells from the same mouse model or human samples are warranted to further confirm the applicability of our findings.

In addition to the effects of PPP1R11 on PP1‐mediated NF‐κB activation, our global transcriptomic analysis of PPP1R11 siRNA‐treated cells confirmed enrichment of the NF‐κB pathway but further revealed other molecules potentially involved in the PPP1R11‐mediated effect on cytokine expression. We like to highlight, however, that all results on mRNA level should always be taken with caution due to the fact that RNA and protein level not necessarily correlate.^55^ To further guide future studies on molecules more likely to be related to a functional protein product, we further provide the reader with classification of the genes into HEG and LEG.^38^ One outstanding HEG from the list of DEGs in PPP1R11‐silenced cells, and confirmed on protein level, was the phosphatase PTPN22. PTPN22 is known to negatively regulate TCR stimulation by dephosphorylating proximal kinases of TCR signaling like the Src family kinases LCK and FYN, and other upstream molecules like CD3 and ZAP‐70.^58^, ^76^, ^77^ Although our results indicate a role of PPP1R11 downstream of PMA/Iono targets and via modulation of PP1, and hence, NF‐κB activity, regulation of PTPN22 may be another mechanism used by PPP1R11 to further modulate T cell activation, perhaps as a secondary effect. Notably, *PTPN22* as well as *NFKB1* genes are strongly associated with multiple autoimmune diseases,^78^, ^79^ and our findings that PPP1R11 modulates T cell resistance to Tregs allow to speculate that in addition to their direct effects in immune cells, susceptibility of T cells to Tregs involving these genes may add to their role in autoimmune disease. Furthermore, since the MAPK, AKT, and NF‐κB pathways crosstalk via several molecules, it is possible that PPP1R11 affects joint signaling molecules between the two pathways. Besides, there have been extensive clinical and experimental reports indicating that the MAPK and AKT signaling pathways are involved in inducing T cell resistance to Tregs.^8^, ^16^, ^17^, ^18^ In light of these findings, we speculate that targeting these pathways by modulating PPP1R11 may be exploited as a novel therapeutic option for autoimmune diseases in the future.

Several kinases and phosphatases have been well‐established drug targets that can be effectively targeted. Drugs targeting kinases are mainly directed toward their catalytic cores. Unlike kinases, substrate specificity of phosphatases is mainly controlled by noncatalytic subunits and other interacting proteins. While immunosuppressive drugs such as cyclosporine A and FK506 have met reasonable successes with direct targeting of the catalytic core of PP2B/calcineurin, similar strategies targeting the catalytic core may elicit unspecific and side effects in the case of PP1. This is because PP1 has numerous subunits and interacting proteins that probably regulate the varying tissue and context‐specific functions of PP1. Hence, therapeutic intervention with a ubiquitous and multifunctional protein like PP1 needs to be done in a more cell type‐specific manner. Biochemical studies to decipher the exact mechanisms behind the effect of PP1 and PPP1R11 on T cell cytokine expression, as well as studies to explore the in vivo relevance of our findings, are still warranted. Yet, our data suggests that PPP1R11 as a novel negative regulator of T cell activation and T cell resistance toward Tregs may be crucial to regulate the T cell‐specific role of PP1. Hence, targeting PPP1R11 instead of the PP1 catalytic core may be beneficial in eliciting specific immunotherapeutic outcomes in treatment of autoimmune and other immune diseases.

## DISCLOSURE

The authors declare no conflict of interest.

## Supporting information

Supporting information

Supporting information
