# Supplementary material for: Phosphatase inhibitor PPP1R11 modulates resistance of human T cells toward Treg‐mediated suppression of cytokine expression
Source: J Leukoc Biol. 2019 Mar 18;106(2):413–30. doi: 10.1002/JLB.2A0618-228R (PMC6850362; doi:10.1002/JLB.2A0618-228R)
Supplement: Supplementary file 1 — Supporting information [file JLB-106-413-s001.pdf]

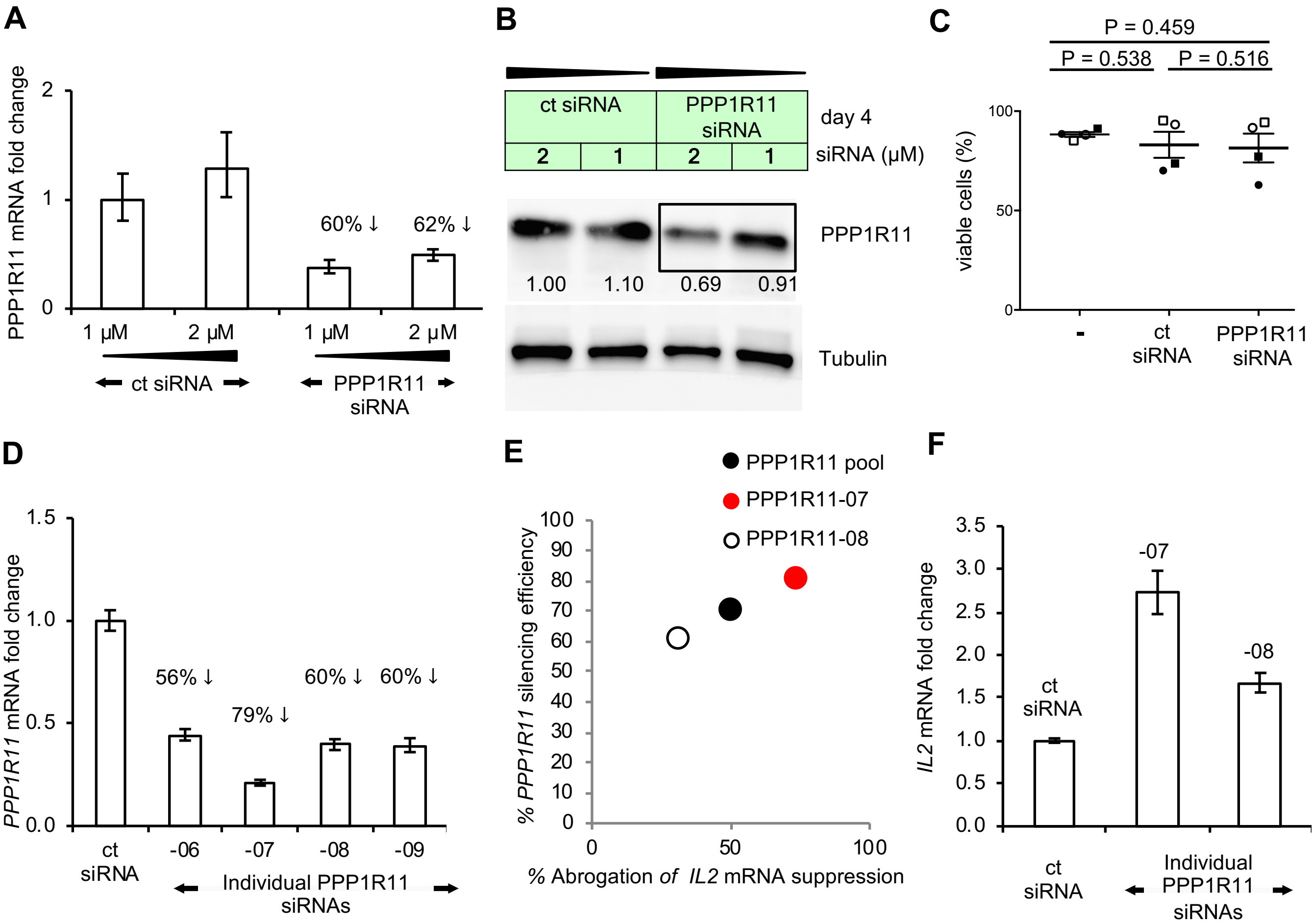

SUPPLEMENTAL FIGURE 1. (A & B): Dose optimization for PPP1R11 siRNA pool. T cells were treated with indicated doses of control siRNA or PPP1R11 pool for (A) 3 days or (B) 4 days. PPP1R11 levels were normalized to (A) GAPDH (mRNA) and (B) Tubulin (protein) measured by (A) qRT-PCR and (B) Western Blot respectively. The results are represented as fold change compared to expression levels in control siRNA treated cells (set to 1) and are representative of 2 donors for both mRNA and protein silencing (mean ± SD for PCR triplicates). Additionally, percentage silencing with respective doses of PPP1R11 siRNAs are represented as compared to respective dose of individual control siRNAs. Higher dose of PPP1R11 siRNA pool (2µM) increased silencing efficiency both in mRNA and protein level hence was chosen for further experiments. **(C)** Cells were transfected with 2 µM of indicated siRNAs for 4.5 days, or left untreated ("-", no transfection). Subsequently, cells were stimulated with plate-bound anti-CD3 and soluble anti-CD28 antibodies for 4.5 days and then stained with viability dye to determine viability by flow cytometry. Percentage of live cells (pre-gated on lymphocytes based on fsc/ssc and after doublet discrimination) is shown for n=4 donors (represented by individual symbols; lines and error bars represent mean ± SEM). P values were determined by paired t test. **(D-F)** De-convolution of the effect of different siRNAs for PPP1R11 silencing. T cells were treated with 2 µM of control siRNA, PPP1R11 siRNA pool (#06 - 09) or individual PPP1R11 siRNAs (#06, 07, 08, 09 as indicated) for 4.5 days. T cells were further stimulated for 3 hours with TCR stimulation. *PPP1R11* and *IL2* mRNA levels were measured by qRT-PCR and normalized to *RPL13A* mRNA. In (D) and (F), the results are represented as fold change compared to expression levels in control siRNA treated cells (set to 1) and figures are representative of 2 donors each (mean ± SD of PCR triplicates). In (E), suppression assay involving Tregs and afore mentioned siRNA-treated T cells were conducted as described in Main Figure 2. Individual silencing efficiencies with tested siRNAs were correlated with observed abrogation of *IL2* mRNA suppression in respective cases (Pearson correlation coefficient = 0.99) (each siRNA represented by a different color).

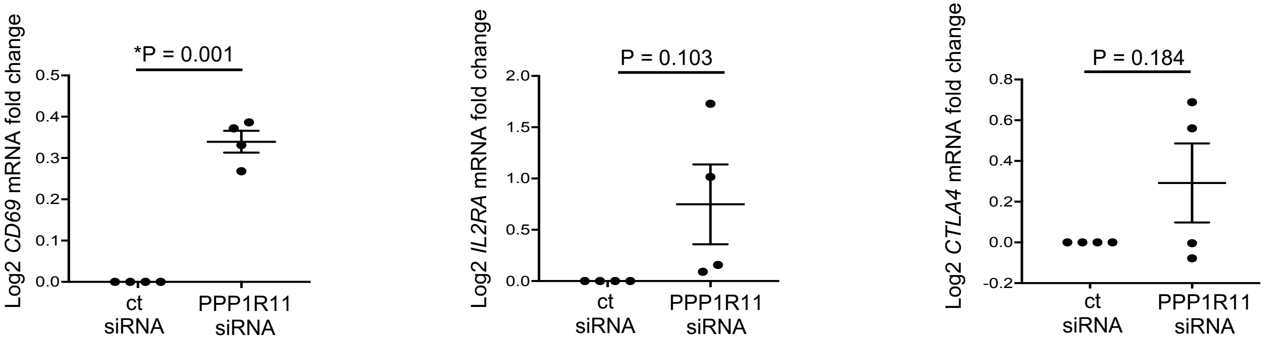

SUPPLEMENTAL FIGURE 2: Effect of PPP1R11 silencing on activation-induced genes. T cells were treated with 2 µM of PPP1R11 siRNA pool or non-targeting (ct) siRNA for 4.5 days and then activated for 3 hours with soluble cross linked anti-CD3/CD28 antibodies at 37˚C. Respective mRNA levels measured by qRT-PCR were normalized to *GAPDH* mRNA and to expression levels in unstimulated cells treated with respected siRNAs. Averaged figures for respective mRNAs (mean ± SEM of 4 donors) are expressed as fold change PPP1R11 siRNA compared with control siRNA (Log2 set to 0). P values were determined by unpaired, one sample, two-sided t test (*P < 0.05) .

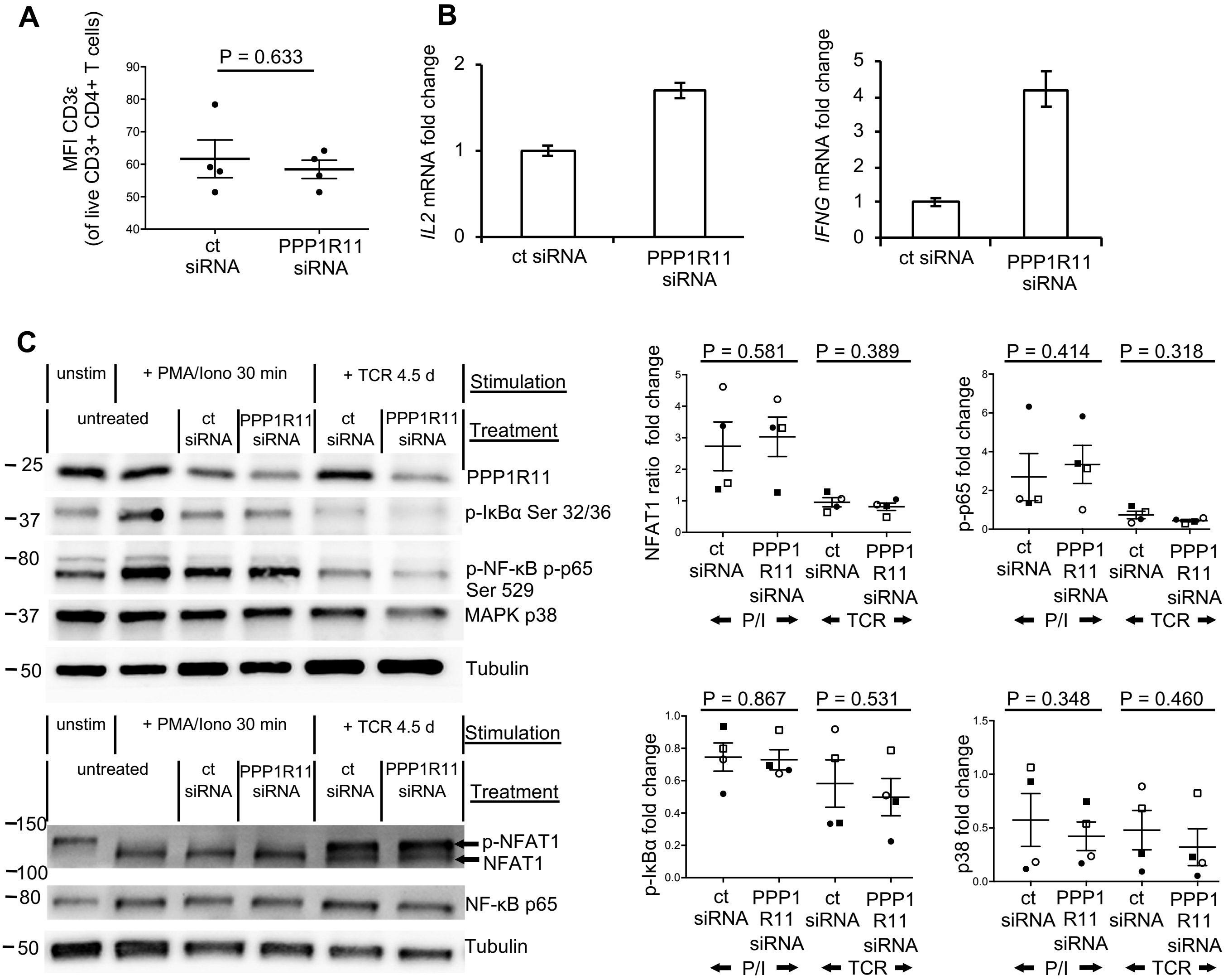

SUPPLEMENTAL FIGURE 3. Studies on the mechanism of PPP1R11-induced cytokine expression in stimulated T cells. **(A)** T cells were treated with 2 μM of PPP1R11 siRNA pool or non-targeting siRNA for 4.5 days and activated for 4.5 days with plate-bound anti-CD3 and soluble anti-CD28 antibodies at 37˚C. CD3ε surface expression was analyzed by surface staining and flow cytometry. Median fluorescence intensity (MFI) for CD3ε is shown, pre-gated on lymphocytes (by fsc/ssc), single cells (by doublet discrimination), live cells (by viability dye) and CD3+CD4+ T cells. Mean ± SEM of 4 donors is shown and P value was determined by paired t test. **(B)** PPP1R11 silencing imparts an activated phenotype to T cells upon PMA/Ionomycin (PMA/Iono) stimulation. T cells were treated with 2 μM of PPP1R11 siRNA pool or non-targeting control siRNA for 4.5 days and activated for 3 hours with PMA/Iono stimulation. Respective cytokine mRNA levels measured by qRT-PCR were normalized to *GAPDH* mRNA and are represented as fold changes compared to expression levels in cells treated with control siRNA (set to 1). Representative expression for *IL2* mRNA (left) and *IFNG* mRNA (right) (mean ± SD of PCR triplicates) for 2 donors is shown. **(C)** Western Blot analysis of canonical TCR signaling molecules in T cells treated with 2 μM of PPP1R11 siRNA pools or non-targeting siRNA for 4.5 days and then activated (i) for 4.5 days with plate-bound anti-CD3 and soluble anti-CD28 antibodies or (ii) for 30 minutes with PMA/Iono at 37˚C. Expression level of several proteins or phospho ("p-") proteins was determined by quantifying Western Blots and normalization to tubulin or corresponding total antibodies. For NFAT1, the ratio of NFAT1/p-NFAT1 was then determined. The normalized values are represented as fold change compared to expression level of the protein in unstimulated T cells (set to 1). The left panel shows blots from a representative donor, and the right panel shows quantification values for n=4 donors (mean ± SEM of 4 donors). P values were determined by paired, two-sided t test.

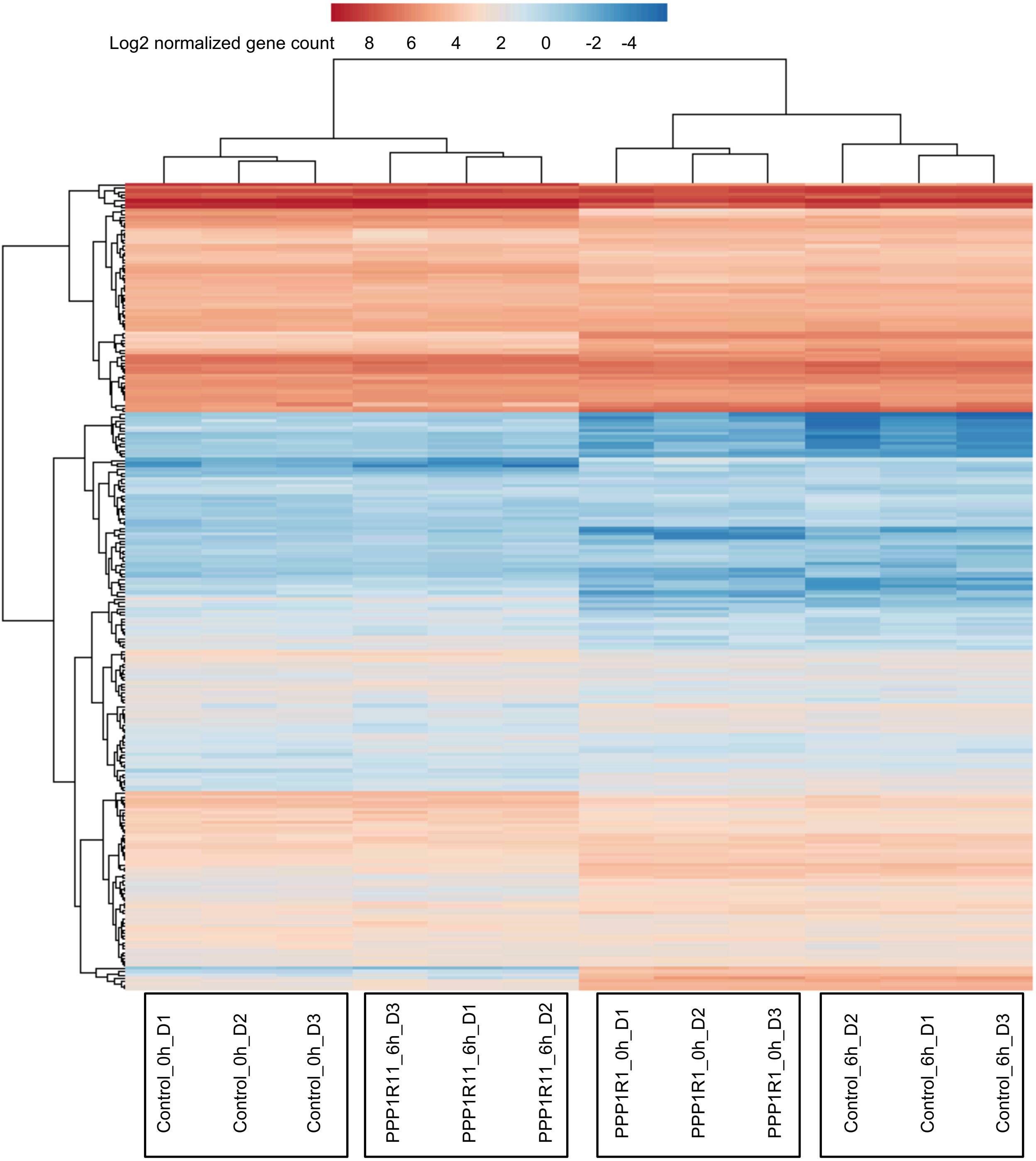

SUPPLEMENTAL FIGURE 4: PPP1R11 silencing enforces a unique gene signature on T cells. The heat map represents the log2 of normalized gene count upon individual siRNA treatments (4.5 days) and, where indicated, subsequent TCR stimulation (6h) for 3 donors (“D1”, “D2”, “D3” = Donor 1, 2 ,3). The magnitude of normalized gene count is represented according to the indicated color scale. Only genes detected in all 3 donors and under all the conditions are included. Clustering indicates complete linkage Euclidian distance.

A

#	Processes	Total	pValue	Min FDR	p-value	FDR	In Data	Network Objects from Active Data
1	nucleoside transport	4	0,0002	0,4335	0,0002	0,4335	3	SLC29A2, ENT1, PMP34
2	regulation of cholesterol metabolic process	49	0,0002	0,4335	0,0002	0,4335	8	CYP51A1, SC5D, SCD, LMF1, PEX2, SREBP2 precursor, ARV1, DHCR7
3	regulation of steroid metabolic process	83	0,0005	0,4867	0,0005	0,4867	10	StARD4, CYP51A1, SC5D, SCD, LMF1, PEX2, SREBP2 precursor, ARV1, DHCR7, INSIG1
4	alcohol biosynthetic process	72	0,0007	0,4867	0,0007	0,4867	9	CYP51A1, SC5D, SC4MOL, HISPPD2A, IMPK, ARV1, DHCR7, INSIG1, DCoHM
5	cholesterol biosynthetic process	34	0,0009	0,4867	0,0009	0,4867	6	CYP51A1, SC5D, SC4MOL, ARV1, DHCR7, INSIG1
6	secondary alcohol biosynthetic process	35	0,0011	0,4867	0,0011	0,4867	6	CYP51A1, SC5D, SC4MOL, ARV1, DHCR7, INSIG1
7	uridine transport	2	0,0012	0,4867	0,0012	0,4867	2	SLC29A2, ENT1
8	positive regulation of cholesterol esterification	2	0,0012	0,4867	0,0012	0,4867	2	StARD4, SCD
9	pyrimidine nucleoside transport	2	0,0012	0,4867	0,0012	0,4867	2	SLC29A2, ENT1
10	sterol biosynthetic process	37	0,0015	0,5489	0,0015	0,5489	6	CYP51A1, SC5D, SC4MOL, ARV1, DHCR7, INSIG1
11	regulation of steroid biosynthetic process	67	0,0020	0,6219	0,0020	0,6219	8	StARD4, CYP51A1, SC5D, SCD, PEX2, SREBP2 precursor, DHCR7, INSIG1
12	lipid biosynthetic process	415	0,0022	0,6219	0,0022	0,6219	26	FATP, CYP51A1, Rab-5A, CERT, ADAS, PI3K class II (CII-alpha), SC5D, SC4MOL, coenzyme Q2 homolog, prenyltransferase (yeast), B3GNT5, CTE2, SGPP1, PLD6, DHRS7B, SCD, PI3K cat class IA (p110-beta), PEX2, DES1, SREBP2 precursor, ARV1, DHCR7, DCTN6, SMS1, INSIG1, PIPKI gamma, PI3K cat class IA (p110-alpha)
13	regulation of cholesterol biosynthetic process	41	0,0025	0,6219	0,0025	0,6219	6	CYP51A1, SC5D, SCD, PEX2, SREBP2 precursor, DHCR7
14	respiratory chain complex III assembly	9	0,0029	0,6219	0,0029	0,6219	3	SGA-81M, UQCRB, C11orf83
15	mitochondrial respiratory chain complex III assembly	9	0,0029	0,6219	0,0029	0,6219	3	SGA-81M, UQCRB, C11orf83
16	organic cyclic compound metabolic process	4032	0,0031	0,6219	0,0031	0,6219	164	FMT, Nibrin, StARD4, CYP51A1, FLJ13149, RPUSD4, 2'-PDE, Spindlin, SLC29A2, SRB7, ZNF800, TRIP11, ATRIP, CCBL2, METTL2A, MGA, PAF49, PIAS3, DCUP, ZN805, ZNF594, TARSL2, APLF, ENT1, UQCRQPC, MOCS3, SC5D, SC4MOL, NDPK A, Twistnb, Axin2, SNFT, ZNF845, UXS1, Suv39H1, POP1 (RNase P/MRP subunit), CTE2, ZNF92, NDUFA8, MafK, CoREST, B3GALNT2, FANCD2, ZNF569, SFRS4, RNASEH2A, PLD6, CGI-09, METTL2B, DMAP1, GRINL1A, HSF2, WDR79, POLE3 (YBL1), JMJD2B, SMC3, SCML2, C17orf42, SCD, ClpX, ETR3, Geminin, DROSHA, UGDH, ZCCHC6, PAPD5, NSUN5B, BMS1 homolog, ZNF367, SURF5, GADD45 alpha, PAF1, ZNF529, PEX2, SMARCA3, ALAS1, ZNF619, BAZ1B, Pinin, MRM1, DUE-B, NDUFS4, NDUFC2, ATF-5, ZNF124, LARP7, CREST, C17orf85, UQCRB, ATP5E, CTR9, ZNF438, SREBP2 precursor, MYT1, KLF5, ZNF250, Gemin7, MJD (ataxin-3), c-Myb, ZNF669, MTF-1, SYHH, ARV1, ZNF264, MMAB, ZNF414, YCL1, DHCR7, Securin, Cep290, SLC19A2, HYPA, SMC5, ZPR1, TAFII28, ZNF295, TCFbeta1, COMT, AP1S1A, SPBP, ZNF776, TRAP100, ELF1, C11orf83, NOC4L, CGI-99, KLF15, Hedls, DGCR14, BLAP75, Midasin, SLTM, TDRD3, Rcor2, CDC5L, HMG20A, TAF13, COX VIIb-1, INSIG1, C1orf124, ZNF354C, DCoHM, RIOK3, WDR37, C20orf29, UBE2W, UTP6, PCGF3, Eftud1, ZNF827, CC2D1A, NCOA6 (TRBP), WDSOF1, APEX2, RBM10, ZNF573, ELF2, RBM13, YT521, COMMD3, ZNF48, CstF64, DDX41, ELL
17	protein quality control for misfolded or incompletely synthesized proteins	30	0,0032	0,6219	0,0032	0,6219	5	N-glycanase 1, MJD (ataxin-3), Klhl15, AP1S1A, UBE2W
18	organic hydroxy compound biosynthetic process	106	0,0034	0,6219	0,0034	0,6219	10	CYP51A1, SC5D, SC4MOL, PEX2, HISPPD2A, IMPK, ARV1, DHCR7, INSIG1, DCoHM
19	nucleoside transmembrane transport	3	0,0034	0,6219	0,0034	0,6219	2	SLC29A2, ENT1
20	regulation of cholesterol esterification	3	0,0034	0,6219	0,0034	0,6219	2	StARD4, SCD
21	mitochondrial fusion	19	0,0035	0,6219	0,0035	0,6219	4	AFG3L2, PLD6, BFL1, FAM73A
22	mitochondrial respiratory chain complex III biogenesis	10	0,0040	0,6684	0,0040	0,6684	3	SGA-81M, UQCRB, C11orf83
23	vasoconstriction	11	0,0054	0,6684	0,0054	0,6684	3	PI3K class II (CII-alpha), ARMET, BBS6
24	tRNA C5-cytosine methylation	4	0,0067	0,6684	0,0067	0,6684	2	METTL2A, METTL2B
25	cholesterol biosynthetic process via lathosterol	4	0,0067	0,6684	0,0067	0,6684	2	SC5D, DHCR7
26	cell proliferation in hindbrain	4	0,0067	0,6684	0,0067	0,6684	2	DSCR2, ATF-5
27	cholesterol esterification	4	0,0067	0,6684	0,0067	0,6684	2	StARD4, SCD
28	maltose metabolic process	4	0,0067	0,6684	0,0067	0,6684	2	GANC, MGAM
29	cholesterol biosynthetic process via desmosterol	4	0,0067	0,6684	0,0067	0,6684	2	SC5D, DHCR7
30	protein import into peroxisome matrix, docking	4	0,0067	0,6684	0,0067	0,6684	2	PEX13, PEX5R
31	steroid esterification	4	0,0067	0,6684	0,0067	0,6684	2	StARD4, SCD
32	sterol esterification	4	0,0067	0,6684	0,0067	0,6684	2	StARD4, SCD
33	cerebellar granule cell precursor proliferation	4	0,0067	0,6684	0,0067	0,6684	2	DSCR2, ATF-5
34	spindle assembly involved in meiosis	4	0,0067	0,6684	0,0067	0,6684	2	Emi1, Golgin-95
35	regulation of genetic imprinting	4	0,0067	0,6684	0,0067	0,6684	2	CTR9, PI3K cat class IA (p110-alpha)
36	cell proliferation in external granule layer	4	0,0067	0,6684	0,0067	0,6684	2	DSCR2, ATF-5
37	organic substance metabolic process	7009	0,0069	0,6684	0,0069	0,6684	263	FMT, Nibrin, StARD4, FATP, CYP51A1, FLJ13149, MRPL23, AFG3L2, RPUSD4, 2'-PDE, FBXW9, Rab-5A, Spindlin, SLC29A2, SRB7, ZNF800, CERT, TRIP11, ATRIP, CCBL2, METTL2A, UBE2H, MGA, ADAS, PAF49, PIAS3, DCUP, ZN805, ZNF594, TARSL2, APLF, Melanotransferrin, PI3K class II (CII-alpha), PDP2, RAB2A, ENT1, UQCRQPC, MOCS3, SLC7A6, ZDHHC23, FAM105B, SC5D, LYPLAL1, MRPS18C, ERO1Lalpha, SC4MOL, Rab12, Cullin 5, NDPK A, KIAA0143, Twistnb, N-glycanase 1, COQ10B, Axin2, FKBP15, coenzyme Q2 homolog, prenyltransferase (yeast), B3GNT5, VRK2, MOBKL1A, SNFT, ZNF845, UXS1, Suv39H1, FLJ10204, SIAE, MTIF2, NAGLU, POP1 (RNase P/MRP subunit), MLCP (reg), CTE2, ZNF92, NDUFA8, MafK, CoREST, B3GALNT2, FANCD2, SGPP1, ZNF569, COP1, SFRS4, RNASEH2A, p42 KKIALRE, PLD6, TMEM5, DHRS7B, CGI-09, DHRS3, GANC, FLJ11078, METTL2B, Synbindin, DMAP1, GRINL1A, HSF2, PEX13, WDR79, POLE3 (YBL1), JMJD2B, SMC3, SCML2, VCIP135, C17orf42, SCD, ClpX, ETR3, Geminin, LMF1, DROSHA, ERK3, UGDH, ZCCHC6, PAPD5, NSUN5B, BMS1 homolog, ZNF367, SURF5, UBXD7, GADD45 alpha, PAF1, LMTK2, PI3K cat class IA (p110-beta), ZNF529, KCTD5, PEX2, USP40, SMARCA3, HECTD1, HISPPD2A, ARTN, PPP1R3D, ALAS1, ZNF619, BAZ1B, Pinin, HECTD3, GGTA1, MRM1, IMPK, MGAM, DUE-B, NDUFS4, NDUFC2, ATF-5, ZNF124, LARP7, TMEM188, CREST, C17orf85, DES1, UQCRB, MRPS12, ATP5E, CTR9, PSMB4, LZK(MAP3K13), ZNF438, SREBP2 precursor, TMEM129, MYT1, KLF5, ANAPC4, Emi1, RNF181, ZNF250, Gemin7, MJD (ataxin-3), Klhl15, eIF5B (IF-2), c-Myb, HADHB, ZNF669, MTF-1, SYHH, GMF, ARV1, HAN11, DCUN1D4, ZNF264, MMAB, ZNF414, YCL1, NAT12, TRAM2, Wdr20a, DHCR7, MLKL, Securin, KIAA1333, Cep290, STK17B, SLC19A2, HYPA, DCTN6, SMC5, ZPR1, MRPS34, TAFII28, MRPL14, ZNF295, TCFbeta1, COMT, AP1S1A, SPBP, PECI, ZNF776, MRPS14, NLP-1, TRAP100, FKBP3, ELF1, C11orf83, NOC4L, CGI-99, SMS1, KLF15, Hedls, PMP34, DGCR14, BLAP75, Midasin, SLTM, BBS10, Golgin-95, TDRD3, Rcor2, CDC5L, HMG20A, TAF13, COX VIIb-1, INSIG1, C1orf124, BBS6, ZNF354C, DCoHM, RIOK3, WDR37, C20orf29, SSTK, UBE2W, UTP6, PCGF3, AMZ2, Eftud1, ZNF827, Napsin A, PIPKI gamma, PI3K cat class IA (p110-alpha), LARP4, TRIM59, CC2D1A, NCOA6 (TRBP), WDSOF1, APEX2, RBM10, ZNF573, ELF2, RBM13, YT521, COMMD3, ZNF48, CstF64, DDX41, ELL
38	ncRNA metabolic process	568	0,0069	0,6684	0,0069	0,6684	31	FMT, RPUSD4, Spindlin, METTL2A, PAF49, TARSL2, MOCS3, Suv39H1, POP1 (RNase P/MRP subunit), PLD6, CGI-09, METTL2B, DROSHA, PAPD5, NSUN5B, BMS1 homolog, MRM1, DUE-B, SYHH, TAFII28, NOC4L, CGI-99, Midasin, TAF13, RIOK3, WDR37, UTP6, WDSOF1, RBM13, CstF64, ELL
39	protein import into peroxisome matrix	12	0,0070	0,6684	0,0070	0,6684	3	PEX13, PEX2, PEX5R
40	microtubule polymerization or depolymerization	36	0,0072	0,6755	0,0072	0,6755	5	MAP7D3, NudE, Emi1, C1orf96, Golgin-95
41	cellular response to misfolded protein	24	0,0084	0,7287	0,0084	0,7287	4	MJD (ataxin-3), Klhl15, AP1S1A, UBE2W
42	cellular aromatic compound metabolic process	3893	0,0108	0,7287	0,0108	0,7287	155	FMT, Nibrin, FLJ13149, RPUSD4, 2'-PDE, Spindlin, SLC29A2, SRB7, ZNF800, TRIP11, ATRIP, CCBL2, METTL2A, MGA, PAF49, PIAS3, DCUP, ZN805, ZNF594, TARSL2, APLF, ENT1, UQCRQPC, MOCS3, NDPK A, Twistnb, Axin2, SNFT, ZNF845, UXS1, Suv39H1, POP1 (RNase P/MRP subunit), CTE2, ZNF92, NDUFA8, MafK, CoREST, B3GALNT2, FANCD2, ZNF569, SFRS4, RNASEH2A, PLD6, CGI-09, METTL2B, DMAP1, GRINL1A, HSF2, WDR79, POLE3 (YBL1), JMJD2B, SMC3, SCML2, C17orf42, ClpX, ETR3, Geminin, DROSHA, UGDH, ZCCHC6, PAPD5, NSUN5B, BMS1 homolog, ZNF367, SURF5, GADD45 alpha, PAF1, ZNF529, SMARCA3, ALAS1, ZNF619, BAZ1B, Pinin, MRM1, DUE-B, NDUFS4, NDUFC2, ATF-5, ZNF124, LARP7, CREST, C17orf85, UQCRB, ATP5E, CTR9, ZNF438, SREBP2 precursor, MYT1, KLF5, ZNF250, Gemin7, MJD (ataxin-3), c-Myb, ZNF669, MTF-1, SYHH, ZNF264, MMAB, ZNF414, YCL1, Securin, Cep290, SLC19A2, HYPA, SMC5, ZPR1, TAFII28, ZNF295, TCFbeta1, COMT, AP1S1A, SPBP, ZNF776, TRAP100, ELF1, C11orf83, NOC4L, CGI-99, KLF15, Hedls, DGCR14, BLAP75, Midasin, SLTM, TDRD3, Rcor2, CDC5L, HMG20A, TAF13, COX VIIb-1, C1orf124, ZNF354C, DCoHM, RIOK3, WDR37, C20orf29, UBE2W, UTP6, PCGF3, Eftud1, ZNF827, CC2D1A, NCOA6 (TRBP), WDSOF1, APEX2, RBM10, ZNF573, ELF2, RBM13, YT521, COMMD3, ZNF48, CstF64, DDX41, ELL
43	regulation of cilium-dependent cell motility	5	0,0109	0,7287	0,0109	0,7287	2	C1orf96, BBS6
44	regulation of cilium beat frequency involved in ciliary motility	5	0,0109	0,7287	0,0109	0,7287	2	C1orf96, BBS6
45	regulation of cilium beat frequency	5	0,0109	0,7287	0,0109	0,7287	2	C1orf96, BBS6
46	regulation of cilium movement involved in cell motility	5	0,0109	0,7287	0,0109	0,7287	2	C1orf96, BBS6
47	regulation of alcohol biosynthetic process	55	0,0109	0,7287	0,0109	0,7287	6	CYP51A1, SC5D, SCD, PEX2, SREBP2 precursor, DHCR7
48	response to misfolded protein	26	0,0111	0,7287	0,0111	0,7287	4	MJD (ataxin-3), Klhl15, AP1S1A, UBE2W
49	heterocycle metabolic process	3881	0,0127	0,7287	0,0127	0,7287	154	FMT, Nibrin, FLJ13149, RPUSD4, 2'-PDE, Spindlin, SLC29A2, SRB7, ZNF800, TRIP11, ATRIP, METTL2A, MGA, PAF49, PIAS3, DCUP, ZN805, ZNF594, TARSL2, APLF, ENT1, UQCRQPC, MOCS3, NDPK A, Twistnb, Axin2, SNFT, ZNF845, UXS1, Suv39H1, POP1 (RNase P/MRP subunit), CTE2, ZNF92, NDUFA8, MafK, CoREST, B3GALNT2, FANCD2, ZNF569, SFRS4, RNASEH2A, PLD6, CGI-09, METTL2B, DMAP1, GRINL1A, HSF2, WDR79, POLE3 (YBL1), JMJD2B, SMC3, SCML2, C17orf42, ClpX, ETR3, Geminin, DROSHA, UGDH, ZCCHC6, PAPD5, NSUN5B, BMS1 homolog, ZNF367, SURF5, GADD45 alpha, PAF1, ZNF529, SMARCA3, ALAS1, ZNF619, BAZ1B, Pinin, MRM1, DUE-B, NDUFS4, NDUFC2, ATF-5, ZNF124, LARP7, CREST, C17orf85, UQCRB, ATP5E, CTR9, ZNF438, SREBP2 precursor, MYT1, KLF5, ZNF250, Gemin7, MJD (ataxin-3), c-Myb, ZNF669, MTF-1, SYHH, ZNF264, MMAB, ZNF414, YCL1, Securin, Cep290, SLC19A2, HYPA, SMC5, ZPR1, TAFII28, ZNF295, TCFbeta1, COMT, AP1S1A, SPBP, ZNF776, TRAP100, ELF1, C11orf83, NOC4L, CGI-99, KLF15, Hedls, DGCR14, BLAP75, Midasin, SLTM, TDRD3, Rcor2, CDC5L, HMG20A, TAF13, COX VIIb-1, C1orf124, ZNF354C, DCoHM, RIOK3, WDR37, C20orf29, UBE2W, UTP6, PCGF3, Eftud1, ZNF827, CC2D1A, NCOA6 (TRBP), WDSOF1, APEX2, RBM10, ZNF573, ELF2, RBM13, YT521, COMMD3, ZNF48, CstF64, DDX41, ELL
50	histone monoubiquitination	27	0,0127	0,7287	0,0127	0,7287	4	SCML2, PAF1, CTR9, PCGF3

B

#	Maps	Total	pValue	Min FDR	p-value	FDR	In Data	Network Objects from Active Data
1	Immune response_Classical complement pathway	16	0,0000	0,0001	0,0000	0,0001	6	C3b, iC3b, C3dg, C3a, C3, C3c
2	Immune response_Lectin induced complement pathway	17	0,0000	0,0001	0,0000	0,0001	6	C3b, iC3b, C3dg, C3a, C3, C3c
3	Immune response_Alternative complement pathway	19	0,0000	0,0001	0,0000	0,0001	6	C3b, iC3b, C3dg, C3a, C3, C3c
4	Alternative complement cascade disruption in age-related macular degeneration	12	0,0001	0,0045	0,0001	0,0045	4	C3b, iC3b, C3a, C3
5	Complement pathway disruption in thrombotic microangiopathy	17	0,0036	0,2569	0,0036	0,2569	3	C3b, C3a, C3
6	Immune response_Generation of memory CD4+ T cells	24	0,0098	0,5129	0,0098	0,5129	3	BFL1, CCR7, L-selectin
7	K-RAS signaling in pancreatic cancer	33	0,0235	0,5129	0,0235	0,5129	3	B-Raf, PDK (PDPK1), PLAU (UPA)
8	Development_Thrombopoietin-regulated cell processes	33	0,0235	0,5129	0,0235	0,5129	3	B-Raf, PDK (PDPK1), Fyn
9	Development_SSTR2 in regulation of cell proliferation	14	0,0277	0,5129	0,0277	0,5129	2	B-Raf, PDK (PDPK1)
10	Apoptosis and survival_nAChR in apoptosis inhibition and cell cycle progression	14	0,0277	0,5129	0,0277	0,5129	2	PDK (PDPK1), Fyn
11	Cell cycle_Sister chromatid cohesion	14	0,0277	0,5129	0,0277	0,5129	2	CHTF18, Securin
12	Development_Leptin signaling via PI3K-dependent pathway	15	0,0316	0,5129	0,0316	0,5129	2	Leptin receptor, PDK (PDPK1)
13	G-protein signaling_Cross-talk between Ras-family GTPases	15	0,0316	0,5129	0,0316	0,5129	2	B-Raf, PDK (PDPK1)
14	Neurophysiological process_Ephrin-B receptors in dendritic spine morphogenesis and synaptogenesis	15	0,0316	0,5129	0,0316	0,5129	2	Fyn, PSD-95
15	Cell adhesion_Integrin inside-out signaling in neutrophils	38	0,0340	0,5129	0,0340	0,5129	3	LTBR1, PDK (PDPK1), L-selectin
16	Development_Transcription regulation of granulocyte development	16	0,0356	0,5129	0,0356	0,5129	2	c-Myb, CD13
17	Chemotaxis_Inhibitory action of lipoxins on IL-8- and Leukotriene B4-induced neutrophil migration	16	0,0356	0,5129	0,0356	0,5129	2	LTBR1, PDK (PDPK1)
18	Immune response_Production and main functions of biologically active leukotrienes and Lipoxin A4	16	0,0356	0,5129	0,0356	0,5129	2	LTBR1, MGST2
19	Immune response_CXCR4 signaling via second messenger	16	0,0356	0,5129	0,0356	0,5129	2	PDK (PDPK1), Fyn
20	Translation_Regulation of translation initiation	16	0,0356	0,5129	0,0356	0,5129	2	PDK (PDPK1), eIF5B (IF-2)
21	Impaired inhibitory action of lipoxins on neutrophil migration in CF	16	0,0356	0,5129	0,0356	0,5129	2	LTBR1, PDK (PDPK1)
22	Development_Melanocyte development and pigmentation	17	0,0399	0,5129	0,0399	0,5129	2	B-Raf, PDK (PDPK1)
23	Role of tumor microenvironment in plexiform neurofibroma formation in neurofibromatosis type 1	17	0,0399	0,5129	0,0399	0,5129	2	Semaphorin 4F, PDK (PDPK1)
24	Immune response_Inhibitory action of lipoxins on superoxide production induced by IL-8 and Leukotriene B4 in neutrophils	17	0,0399	0,5129	0,0399	0,5129	2	LTBR1, PDK (PDPK1)
25	Impaired inhibitory action of lipoxins on ROS production in neutrophils in CF	18	0,0444	0,5129	0,0444	0,5129	2	LTBR1, PDK (PDPK1)
26	Neurophysiological process_Constitutive and regulated NMDA receptor trafficking	19	0,0490	0,5129	0,0490	0,5129	2	Fyn, PSD-95
27	G-protein signaling_TC21 regulation pathway	19	0,0490	0,5129	0,0490	0,5129	2	B-Raf, TC21
28	Immune response_C5a signaling	19	0,0490	0,5129	0,0490	0,5129	2	B-Raf, PDK (PDPK1)
29	Immune response_LTBR1 signaling	19	0,0490	0,5129	0,0490	0,5129	2	LTBR1, PDK (PDPK1)
30	Chemotaxis_Lipoxin inhibitory action on Formyl-Met-Leu-Phe-induced neutrophil chemotaxis	20	0,0538	0,5129	0,0538	0,5129	2	LTBR1, PDK (PDPK1)
31	Immune response_C3a signaling	20	0,0538	0,5129	0,0538	0,5129	2	KLF5, C3a
32	Immune response_PD-1-induced metabolic changes in T cells	20	0,0538	0,5129	0,0538	0,5129	2	PDK (PDPK1), Fyn
33	Cell cycle_Role of APC in cell cycle regulation	20	0,0538	0,5129	0,0538	0,5129	2	Geminin, Securin
34	Development_Beta-adrenergic receptor-induced regulation of ERK	20	0,0538	0,5129	0,0538	0,5129	2	B-Raf, PDK (PDPK1)
35	Linoleic acid metabolism	3	0,0557	0,5129	0,0557	0,5129	1	PA2G6
36	Development_Adenosine A2A receptor signaling	22	0,0638	0,5129	0,0638	0,5129	2	B-Raf, PDK (PDPK1)
37	FGF signaling in pancreatic cancer	22	0,0638	0,5129	0,0638	0,5129	2	PDK (PDPK1), PLAU (UPA)
38	Development_VEGF signaling via VEGFR2 - generic cascades	49	0,0642	0,5129	0,0642	0,5129	3	PDK (PDPK1), PLAU (UPA), Fyn
39	Immune response_Regulation of T cell function by CTLA-4	23	0,0691	0,5129	0,0691	0,5129	2	PDK (PDPK1), Fyn
40	Immune response_iC3b-induced phagocytosis via alpha-M/beta-2 integrin	23	0,0691	0,5129	0,0691	0,5129	2	PI4K2B, iC3b
41	Apoptosis and survival_NO synthesis and signaling	23	0,0691	0,5129	0,0691	0,5129	2	PDK (PDPK1), PSD-95
42	Acetylcholine biosynthesis and metabolism	4	0,0736	0,5129	0,0736	0,5129	1	PA2G6
43	Neurophysiological process_nNOS signaling in neuronal synapses	4	0,0736	0,5129	0,0736	0,5129	1	PSD-95
44	Anandamide biosynthesis and metabolism	4	0,0736	0,5129	0,0736	0,5129	1	PTPN22
45	Immune response_M-CSF-receptor signaling pathway	52	0,0741	0,5129	0,0741	0,5129	3	PDK (PDPK1), PLAU (UPA), Fyn
46	Development_Growth factors in regulation of oligodendrocyte precursor cell survival	24	0,0745	0,5129	0,0745	0,5129	2	PDK (PDPK1), Fyn
47	Development_NCAM1-mediated neurite outgrowth, synapse assembly and neuronal survival	24	0,0745	0,5129	0,0745	0,5129	2	Fyn, PSD-95
48	Development_Membrane-bound ESR1: interaction with growth factors signaling	24	0,0745	0,5129	0,0745	0,5129	2	B-Raf, PDK (PDPK1)
49	Immune response_IL-7 signaling in T lymphocytes	24	0,0745	0,5129	0,0745	0,5129	2	PDK (PDPK1), Fyn
50	Development_PEDF signaling	25	0,0800	0,5129	0,0800	0,5129	2	PDK (PDPK1), BFL1

SUPPLEMENTAL FIGURE 5: Enrichment analyses of RNA-seq data. **(A)** Enrichment analysis report from comparative gene ontology (GO) analysis by Process Networks. GO analysis was performed with MetaCore software. Included in the analysis were differentially expressed genes upon PPP1R11 siRNA treatment with control siRNA treatment at 0h ($P < 0.05$). Top 50 process networks were represented from the Metacore analysis. **(B)** Enrichment analysis report from comparative gene ontology (GO) analysis by Pathway Maps. GO analysis was performed with MetaCore software. Included in the analysis were genes whose stimulation induced expression was differentially regulated by PPP1R11 siRNA treatment as compared to control siRNA treatment (referred as interaction term) ($P < 0.05$). Top 50 pathway maps were represented from the Metacore analysis.

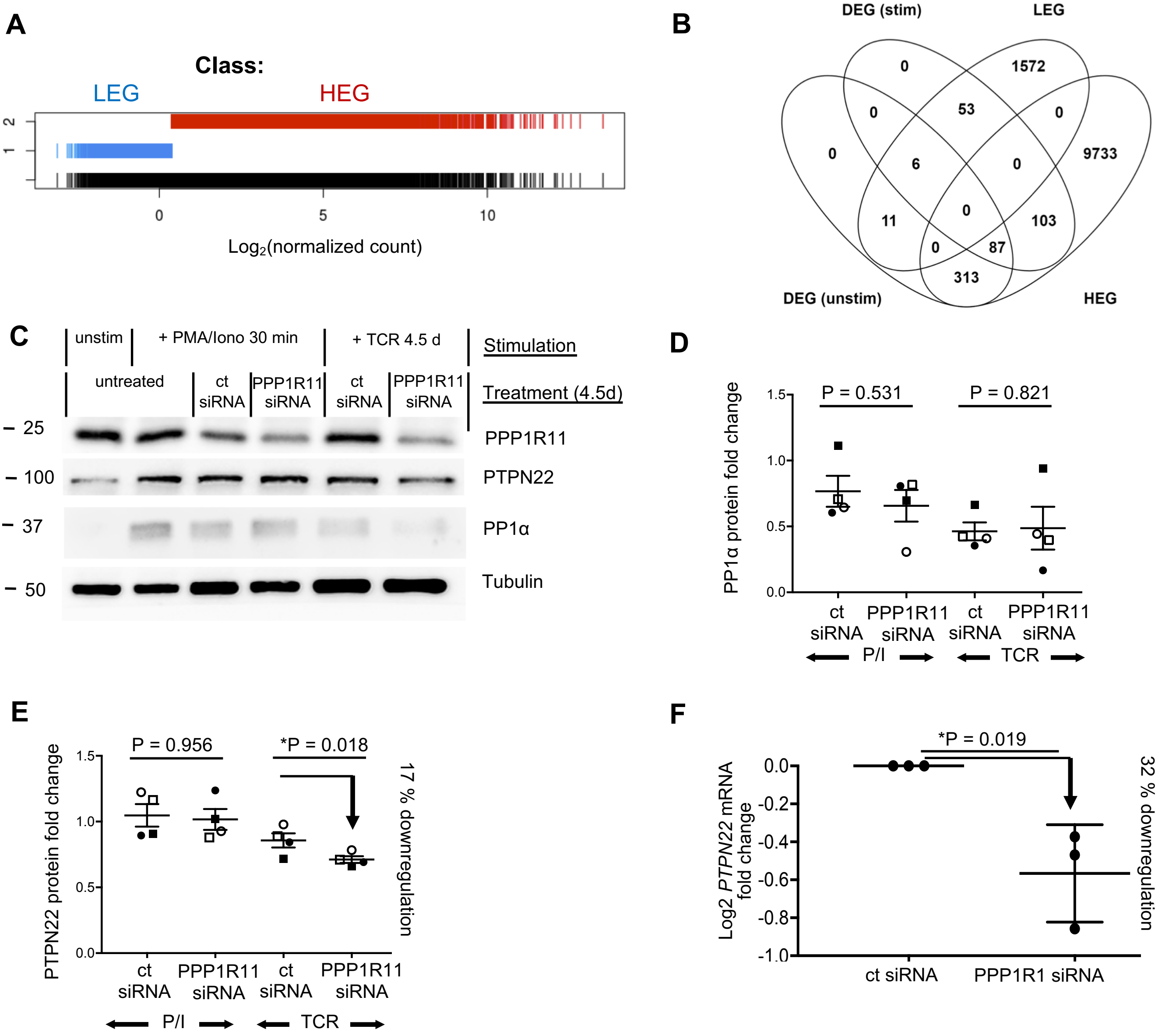

SUPPLEMENTAL FIGURE 6: Protein level validation experiments for RNA-seq data. **(A)** Genes from RNA-seq data were classified into highly expressed genes (HEG) and lowly expressed genes (LEG) (left panel; cluster "1" and "2" respectively), based on the expression (Log_2(normalized count)) mean of all samples. The cutoff for being classified HEG was determined to be 0.38 (Log_2(normalized count)). **(B)** DEGs from Figure 4A ("unstim") and Figure 4B ("stim") were analyzed regarding their expression level, and the overlap with HEG and LEG class genes is shown in the Venn diagram (numbers represent number of genes). **(C-E)** T cells were treated with 2 µM of PPP1R11 siRNA pools or non-targeting siRNA for 4.5 days and then activated either with PMA/Ionomycin for 30 min or with TCR stimulation for 4.5 days at 37˚C. Expression level of several proteins, measured by quantifying Western Blots, were normalized to tubulin and are represented as fold change compared to expression level of the protein in unstimulated T cells (set to 1). (C) is a representative blot and (D & E) represent averaged and individual expression levels for 4 donors (mean ± SEM of 4 donors). P values were determined by paired, two-sided Student's t test. **(F)** Averaged figures for *PTPN22* mRNA level (mean ± SEM of 3 donors) are expressed as log2 transformed fold change compared to control siRNA (log2 set to 0) from the RNA-seq study from Main Figure 4. P value was determined by unpaired, one sample, two-sided t test.
